# Epigenetic Priming by Hypomethylation Enhances the Immunogenic Potential of Tolinapant in T-cell Lymphoma

**DOI:** 10.1158/2767-9764.CRC-23-0415

**Published:** 2024-06-06

**Authors:** George A. Ward, Zhiqiang Zhang, Simone Jueliger, Ilya S. Potapov, Matthew P. Davis, Adam R. Boxall, Jason Taylor, Harold Keer, Andrea Biondo, John F. Lyons, Martin Sims, Tomoko Smyth

**Affiliations:** 1 Astex Pharmaceuticals, Cambridge, United Kingdom.; 2 Astex Pharmaceuticals, Inc., Pleasanton, California.

## Abstract

Programmed cell death mechanisms are important for the regulation of tumor development and progression. Evasion of and resistance to apoptosis are significant factors in tumorigenesis and drug resistance. Bypassing apoptotic pathways and eliciting another form of regulated cell death, namely necroptosis, an immunogenic cell death (ICD), may override apoptotic resistance. Here, we present the mechanistic rationale for combining tolinapant, an antagonist of the inhibitor of apoptosis proteins (IAP), with decitabine, a hypomethylating agent (HMA), in T-cell lymphoma (TCL). Tolinapant treatment alone of TCL cells *in vitro* and in syngeneic *in vivo* models demonstrated that ICD markers can be upregulated, and we have shown that epigenetic priming with decitabine further enhances this effect. The clinical relevance of ICD markers was confirmed by the direct measurement of plasma proteins from patients with peripheral TCL treated with tolinapant. We showed increased levels of necroptosis in TCL lines, along with the expression of cancer-specific antigens (such as cancer testis antigens) and increases in genes involved in IFN signaling induced by HMA treatment, together deliver a strong adaptive immune response to the tumor. These results highlight the potential of a decitabine and tolinapant combination for TCL and could lead to clinical evaluation.

**Significance:**

The IAP antagonist tolinapant can induce necroptosis, a key immune-activating event, in TCL. Combination with DNA hypomethylation enhances tolinapant sensitivity and primes resistant cells by re-expressing necrosome proteins. In addition, this combination leads to increases in genes involved in IFN signaling and neoantigen expression, providing further molecular rationale for this novel therapeutic option.

## Introduction

Dying cancer cells are crucial for antitumor immunity because they stimulate the cross-priming of CD8^+^ T cells (1) which drives the cancer-immunity cycle (2). Therefore, understanding how to invoke immunogenic forms of cell death (ICD), such as necroptosis, using therapeutic compounds is a goal for successful immunotherapy (3–5). Cancer cells employ epigenetic mechanisms to evade certain forms of cell death (6). Furthermore, epigenetic modifications of immune cell effectors can affect immune cell subsets to invoke a more robust adaptive response (7).

Recent studies have demonstrated that when inhibitor of apoptosis proteins (IAP) are absent and under conditions in which there is resistance to apoptosis, a different cell death–inducing complex (the necrosome) can form, leading to necroptosis, a highly immunogenic form of cell death (8). Tolinapant (ASTX660), a potent non-peptidomimetic antagonist of cIAP1, cIAP2, and XIAP (9, 10), has been shown to induce an immunogenic (necroptotic) form of cell death and elicit further immunomodulatory effects in preclinical models of T-cell lymphoma (TCL; ref. 11). In an ongoing phase II trial (NCT02503423), tolinapant showed activity against extensively pretreated peripheral and cutaneous T-cell lymphomas (PTCL and CTCL; refs. 12, 13) and patient samples from this trial have demonstrated some preliminary evidence of immune modulation (11).

However, it is also evident that cancer cells acquire resistance to ICD through multiple mechanisms (14). Several key components of the programmed cell death pathway are regulated by promoter methylation in various cancers (6). Epigenetic reprogramming to reverse this resistance, for example, using DNA hypomethylating agents (HMA), has been proposed (15). RIPK3, a key component of the necrosome, is frequently silenced via methylation in various cancer cell lines. Silencing *RIPK3* has been implicated in therapy resistance in mesothelioma (16) and breast cancer models (17). Re-expression of RIPK3 can be achieved by HMA treatment (17). In addition, low IFN signaling limits the levels of MLKL (18, 19), another key effector of necroptosis. Enhancement of chemokines and cytokines suggests that HMAs have the potential to prime tumor cells toward immunogenic cell death, as well as modulate the tumor microenvironment (7).

Clinical responses to HMAs, such as 5-azacytidine (NCT03593018) and guadecitabine (SGI-110) (#ACTRN12618000028202), have been reported in some subsets of PTCL (20, 21), suggesting that a reduction in methylation may affect PTCL pathology. A phase I/II study investigating the combination of tolinapant and ASTX727 (oral decitabine and cedazauridine; ref. 22) was recently commenced in patients with relapsed/refractory PTCL (NCT05403450).

Here, we investigated the potential of HMA-induced reversal of necroptosis resistance owing to epigenetic silencing and altered cell signaling to promote ICD driven by tolinapant treatment in preclinical TCL models. To this end, we generated engineered TCL cell lines, including caspase-8 knockout (KO) cells, to confirm the role of necroptosis. We also demonstrated that elevation of plasma IP-10 (CXCL10), a key chemoattractant for activated CD8^+^ T cells that was detected in our preclinical models, was observed in patients with PTCL after tolinapant treatment.

## Materials and Methods

### Reagents

The lactate salt of tolinapant (ASTX660) was synthesized using a previously described chemical route (9). Necrostatin-1 (Nec-1; catalog no. N9037, RRID:SCR_000488) was purchased from Sigma. Mouse TNFα (catalog no. 410-MT) was purchased from R&D Systems. Decitabine [5-aza-2′-deoxycytidine (DAC)] was purchased from Sigma. The pan-caspase inhibitor Z-VAD-FMK (catalog no. ALX-260-020-M005) was purchased from Enzo Life Sciences (UK) Ltd.

### Cell Lines

BW5147.G.1.4 (catalog no. TIB-48, RRID:CVCL_6315; BW5147), HH (catalog no. CRL-2105, RRID:CVCL_1280), H9 (catalog no. HTB-176, RRID:CVCL_1240), HuT-78 (catalog no. TIB-161, RRID:CVCL_0337), HuT-102 (catalog no. TIB-162, RRID:CVCL_3526), MJ (catalog no. CRL-8294, RRID:CVCL_1414), and CT26.WT (catalog no. CRL-2638, RRID:CVCL_7256; CT26) cells were obtained from the ATCC. Sup-T1 (catalog no. ACC-140, RRID:CVCL_1714) and Sup-M2 (catalog no. ACC-509, RRID:CVCL_2209) cells were obtained from the German Collection of Microorganisms and Cell Cultures (DSMZ; Braunschweig, Germany). EL4 (catalog no. 85023105, RRID:CVCL_0255) and Karpass-299 (catalog no. 06072604, RRID:CVCL_1324) cells were obtained from the European Collection of Authenticated Cell Cultures (ECACC, Salisbury, UK). All cells were cultured in the medium recommended by the suppliers and supplemented with 10% heat-inactivated FBS at 37°C in a humidified atmosphere of 5% carbon dioxide. All the culture media and supplements were purchased from Thermo Fisher Scientific (Gibco). All cell lines were passaged for no more than 6 months (or 30 passages) after authentication by the cell bank (short tandem repeat PCR) and were routinely screened for *Mycoplasma* (MycoAlert, Lonza).

### *In Vitro* Viability Assays

Cell viability was measured using CellTiter Glo (CTG, Promega). Cells were seeded into 96-well plates one day before compound treatment. Compounds were dissolved in DMSO at a final concentration of 1,000 × and diluted 1:10 in serum-free medium before addition to the cells at 1:100 dilution. The plates were incubated for 3 days, after which the CTG reagent was added according to the manufacturer's protocol. Luminescence was measured using a Hidex Sense Beta Microplate Reader (Hidex). After background subtraction, each well value was normalized as a percentage of the mean DMSO control value and was expressed as a percentage. Sigmoidal dose–response (variable slope) curve fit and IC_50_ values were generated using Prism GraphPad Software (RRID:SCR_002798).

A combination matrix screen of tolinapant and decitabine was conducted in a panel of five human TCL cell lines: HH, H9, HuT-78, HuT-102, and MJ (ChemPartner). Viability was determined using the CTG assay after 3 days. The degree of synergy was calculated using the highest single agent (HSA) method using the Combenefit software (ref. 23; CRUK, Cambridge, UK).

### Live-cell Imaging

Induction of cell death was measured in real time using an IncuCyte S5 live cell imager (Sartorius Ltd.). Cells were incubated with compound in 0.1% (v/v) DMSO for 1–3 days and live images were taken every 3 hours using a 10 × objective. The IncuCyte software was used to calculate the mean confluency from four nonoverlapping phase-contrast images of each well. Cells undergoing lytic cell death were measured by detecting the uptake of Cytotox-NIR reagent (Sartorius Ltd.), which is designed to penetrate cells with a permeabilized plasma membrane, such as those undergoing necroptosis.

### Western Blotting

Cell or tumor lysates were resolved by SDS-PAGE and immunoblotted as described previously (10) with primary antibodies for Western blotting, as listed in Supplementary Table S1. Specific binding was detected using the IR800CW donkey anti-goat (catalog no. 926-32214, RRID:AB_621846), goat anti-rat (catalog no. 926-32219, RRID:1850025), donkey anti-mouse (catalog no. 926-32212, RRID:621847), or anti-rabbit (catalog no. 926-32211, RRID:621843) LI-COR secondary antibodies and an Odyssey Infrared Imaging System (LI-COR).

### qPCR TaqMan Analysis

RNA was extracted from cells and tumors using the RNeasy Mini Kit (#74104, Promega) with on-column DNase digestion using the RNase-Free DNase Set (#79254, Qiagen). A two-step RT-PCR reaction was performed using an Applied Biosystems kit (catalog no. N8080234; Applied Biosystems) was used to generate cDNA. For the first reverse transcription step, a fixed concentration of RNA (1 µg) was mixed with 1 µL oligo dT and 1 µL random hexamers and heated at 65°C for 5 minutes. In the second step, a master mix (2 µL 10X RT buffer, 1.4 µL MgCl_2_, 4 µL dNTP mix, 1 µL RNase inhibitor, 1 µL Multiscribe RT Enzyme, and RNAse-free water) was added to the mixture from step 1, and the reaction was incubated for 30 minutes at 37°C, followed by 95°C for 5 minutes and then cooled to 10°C. The resultant cDNA mixture was diluted 10x with nuclease-free water before performing Taqman qPCR.

Taqman Gene Expression assays (Applied Biosystems) were used for qPCR. *18S* Ribosomal RNA was used as a housekeeping gene. For each reaction, 8 µL of cDNA was mixed with 1 µL of Taqman Gene Expression Assay probe, 1 µL of Taqman *18S* housekeeping control primer (Thermo Fisher Scientific—for primer details, see Supplementary Table S2), and S10 µL of Taqman Gene Expression Master Mix (2X; Applied Biosystems #4444557) in a final volume of 20 µL. Each sample was run in duplicate on the ViiA 7 Real-Time PCR system (Applied Biosystems) under the following conditions: initial activation at 95°C for 20 seconds, followed by 40 cycles of 95°C for 1 second and 60°C for 20 seconds.

### Generation of CRISPR KO and CRISPR Activation Cell Line Clones

CRISPR KO and CRISPR activation (CRISPRa) cells were generated as described in Supplementary Table S3.

### Meso Scale Discovery Cytokine Analysis

Human and mouse cytokine levels were measured using a U-Plex Cytokine 10-spot meso scale discovery (MSD) plate (mouse, catalog no. K15069L-1, human catalog no. K15067L-1, MSD), according to the manufacturer's protocol. The plates were then read using a QuickPlex SQ 120 MSD plate reader. The average blank electrical chemiluminescence values were subtracted from each duplicate for each sample. These values were quantified relative to a standard curve of the calibrator standard using linear regression analysis.

### Luminex Analysis Explorer MAP

Mouse plasma and cell supernatant cytokines were measured using the Rodent MAP 4.0 (Analysis of 42 analytes; CCL6, CRP, Eotaxin, GCP-2, GMCSF, IFNβ, IFNγ, IL1α, IL1 beta, IL2, IL4, IL5, IL6, IL9, IL10, IL12p40, IL12p70, IL17A, IL18, IL23, IL27, IL28, Insulin, IP-10, KC/GRO, MCP-1, MCSF1, MDC, MIP-1α, MIP-1β, MIP-1γ, MMP-9, NGAL, RAGE, PAI-1, SCF, TIMP-1, TNFα, TPO, TSLP, VCAM-1, VEGF-A) on the Luminex platform (Ampersand Biosciences).

### HMGB1 ELISA

Cells were seeded into 96-well plates at 0.5 × 10^6^ cells/mL in fresh medium and treated with various concentrations of tolinapant in the presence or absence of decitabine for 24 hours. HMGB1 levels in the supernatant were measured using an HMGB1 ELISA kit (Tecan, catalog no. ST51011) according to the manufacturer's protocol. Mouse plasma samples were stored as frozen aliquots at −80°C prior to analysis. An aliquot was thawed on ice and analyzed using HMGB1 ELISA, as described above.

### Promoter Methylation Analysis by Bisulfite Modification and Pyrosequencing

TCL cell lines were treated with various concentrations of decitabine for 1–4 days, washed, and harvested. Bisulfite pyrosequencing was conducted at EpigenDx. A bespoke assay was used for the human *RIPK3* promoter region, with primers designed around the known *RIPK3* promoter CpG islands (see Supplementary Fig. S4A). Long interspersed element-1 (LINE-1) primers were used to detect changes in genome-wide methylation.

### Mouse Tumor Models

The care and treatment of animals were in accordance with the United Kingdom Coordinating Committee for Cancer Research guidelines and the United Kingdom Animals (Scientific Procedures) Act 1986 (24, 25). The study protocols were approved by the Agenda Resource Management Ethical Review Committee. AKR/J (The Jackson Laboratory, RRID:IMSR_JAX:000648) and C57BL/6J mice (Charles River Laboratories, RRID:IMSR_JAX:000664) were used to generate the syngeneic TCL models. All mice were used at the age of 7–11 weeks. Two million cancer cells were subcutaneously implanted into the flanks of mice. Treatment was initiated 4 days after cell implantation. Karpas-299 xenografts were prepared by subcutaneously implanting 5 × 10^6^ cells suspended in a 1:1 mixture of PBS and Matrigel into the flanks of CB17 SCID mice (Envigo, RRID:IMSR_RJ:CB17-SCID). Tolinapant was dissolved in water and administered via oral gavage at 25 mg/kg (syngeneic model) or 16 mg/kg (xenograft) once daily during the indicated treatment periods. Decitabine was dissolved in saline and injected intraperitoneally (0.3 mg/kg once daily for 3 days). Both drugs were administered at a dose of 10 mL/kg. The control group received saline as a vehicle control for decitabine and water for tolinapant.

### Pharmacodynamic Studies

Tumor-bearing animals (see above) were used for the pharmacodynamic studies. Tumors were excised at specific timepoints postdose, immediately snap-frozen in liquid nitrogen, and stored at −80°C. Tumor lysates were prepared by grinding the frozen tissue to a fine powder with a mortar and pestle under liquid nitrogen, followed by the addition of ice-cold lysis buffer (1% Triton X-100, 150 mmol/L NaCl, 20 mmol/L Tris·HCl, pH 7.5) containing Complete Mini protease inhibitors (catalog no. 11836153001, Roche) and phosphatase inhibitors (PhosSTOP, catalog no. 04 906 837 001, Roche). Well-mixed samples were incubated on ice for 30 minutes. Lysates were cleared by centrifugation, and protein levels in the supernatant were determined by bicinchoninic acid assay (Pierce) and then normalized. Each sample was mixed with SDS sample buffer and DTT (final concentration of 50 mmol/L), boiled, and analyzed by Western blotting as described above. Blood samples were collected via superficial venepuncture, and plasma was prepared by centrifugation.

### IHC Analysis

Multiplex immunofluorescence analysis of tumor sections (cut from formalin-fixed and paraffin-embedded tissue samples) taken at designated timepoints was performed using a 3-plex OPAL kit in the Leica Bond Rx platform (Propath) using the following antibodies: anti-mouse CD4(Abcam, catalog no. AB288724), anti-mouse CD8 (Abcam, catalog no. AB217344) with the isotype control (Abcam, catalog no. AB172730-1001) for both. Whole slide scans were taken with a Phenocycler Fusion.

### Analysis of Clinical Study Samples

Plasma from subjects enrolled in the phase I–II Study of the Safety, Pharmacokinetics, and Preliminary Activity of ASTX660 in Subjects with Advanced Solid Tumors and Lymphomas (NCT02503423) was collected at screening and on treatment. All the participants provided written informed consent for their samples to be stored and used for research purposes. The study protocol was approved by the Institutional Review Board or independent ethics committee prior to study initiation. Cytokine analysis was performed using the Human ExplorerMAP v. 1.0 (Myriad RBM), as described previously (11). Samples from the first two cycles of 63 patients with PTCL were analyzed. Samples taken pretreatment or up to 2 hours posttreatment were grouped as “early” and used as the baseline. Posttreatment samples, grouped as “late,” were taken on day 2 (a day after the first dose), day 7, day 8, or day 9 at the end of seven daily doses in cycle 1 (C1). Cycle 2 was initiated 7 days after the last tolinapant administration of C1. Statistical analysis used a binomial one-tailed test, and *P* values of 0.05 were deemed significant.

### Data Availability

All data relevant to the study are included in the article or uploaded as Supplementary Data.

## Results

### Tolinapant Treatment Induces Necroptosis in TCL Cell Lines When RIPK3 and MLKL are Expressed to Form the Intact Necrosome

**FIGURE 1:**
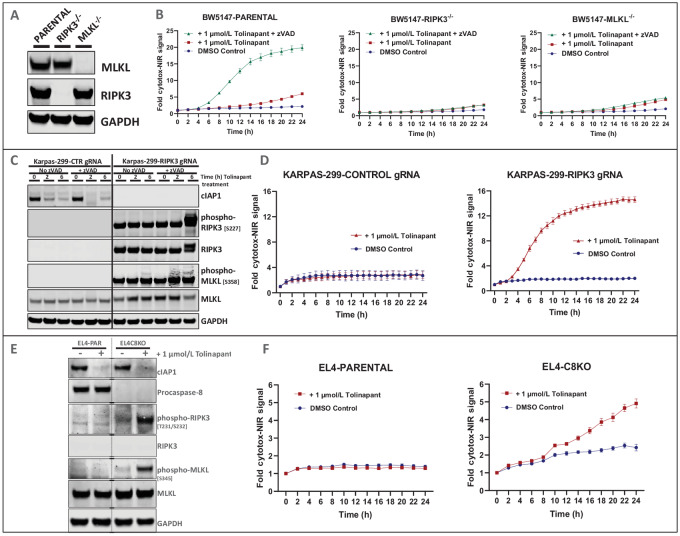
Effect of necrosome protein expression in TCL cell lines on tolinapant-induced cell death. **A,** Western blots of parental BW5147, *RIPK3^−^^/^^−^* BW5147 and *MLKL^−^^/^^−^* BW5147 cell lysates (untreated). **B,** Cytotox-NIR signal captured by real-time microscopy (IncuCyte), detecting membrane permeabilization during lytic cell death after treatment of parental BW5147, *RIPK3^−^^/^^−^* BW5147 and *MLKL^−^^/^^−^* BW5147 cells with tolinapant in the absence or presence of zVAD. **C,** Western blots of Karpas-299 cells transduced with control gRNA (KARPAS-299-CTR gRNA) or with RIPK3 gRNA (KARPAS-299-RIPK3 gRNA) constructs treated with tolinapant ± zVAD for 0, 2, or 6 hours. **D,** Real-time microscopy (IncuCyte) measurement of membrane permeabilization by measuring Cytotox-NIR signal after treatment of KARPAS-299-CTR gRNA or KARPAS-299-RIPK3 gRNA cells with tolinapant. **E,** Western blots of EL4-PAR and *CASP8* KO EL4 (EL4-C8KO) cell lysates after 24-hour treatment with tolinapant. **F,** Real-time microscopy (IncuCyte) measurement of membrane permeabilization by measuring Cytotox-NIR signal after treatment of EL4-PAR or EL4-C8KO cells with tolinapant.

Previously, we demonstrated that the mouse TCL cell line, BW5147.G.1.4 (BW5147), was sensitive *in vitro* and *in vivo* to tolinapant treatment as a single agent and that this response involved necroptosis (11). To further demonstrate the roles of RIPK3 and MLKL, we knocked out each individually in BW5147 cells and compared their responses with tolinapant treatment. Clones of both KO cell lines showed reduced levels of lytic cell death, as measured by the uptake of Cytotox Red dye, compared with the parental cells *in vitro* (Fig. 1A and B; Supplementary Fig. S1A and S2A). These KO cells produced less IL2 and TNFα after treatment with tolinapant in the presence of the caspase inhibitor, zVAD (Supplementary Fig. S2B).

The effects of increased RIPK3 expression were investigated using CRISPRa in Karpas-299 cells. In this human TCL cell line, which is known to have low basal expression of RIPK3 (DepMap portal, RRID:SCR_017655), the expression of endogenous RIPK3 was successfully induced by CRISPRa, and its phosphorylation was further enhanced by tolinapant treatment (Fig. 1C). This was also accompanied by an increase in phospho-MLKL levels (Fig. 1C; Supplementary Fig. S1B). Tolinapant treatment led to significant loss of viability (Supplementary Fig. S2C) and increased lytic cell death in RIPK3-expressing Karpas-299 cells (Fig. 1D). Upregulated RIPK3 expression in Karpas-299 cells also led to increased levels of the proinflammatory cytokines TNFα and IL8 upon tolinapant treatment (Supplementary Fig. S2D).

Caspase-8 KO (C8KO) in the EL4 mouse TCL cell line led to upregulation of both phospho-RIPK3 and phospho-MLKL upon treatment with tolinapant alone (Fig. 1E). This effect was potentiated by adding TNFα to the caspase-8-KO EL4 (EL4-C8KO) cells (Supplementary Fig. S1C). Furthermore, tolinapant treatment led to an increased induction of lytic cell death in the EL4-C8KO cell line compared with that in the parental EL4 (EL4-PAR) cell line (Fig. 1F). Nec-1 treatment rescued the decrease in viability observed in EL4-C8KO cells (Supplementary Fig. S2E). The EL4-C8KO cell line produced more IL2 and TNFα upon treatment with tolinapant than the parental line (Supplementary Fig. S2F). Necroptosis biomarkers were elevated in EL4-C8KO cells following treatment with tolinapant (Supplementary Fig. S3A), decitabine (Supplementary Fig. S3B), or a combination of the two (Supplementary Fig. S3B). Elevated levels of genes involved in IFN signaling were observed in EL4-C8KO cells after decitabine treatment (Supplementary Fig. S3B). Increased lytic cell death was measured after treatment of EL4-C8KO cells with the decitabine plus tolinapant combination compared with the EL4-PAR line (Supplementary Fig. S3C).

### HMA Treatment Restores Expression Levels of Key Necrosome Components in TCL Cell Lines

**FIGURE 2:**
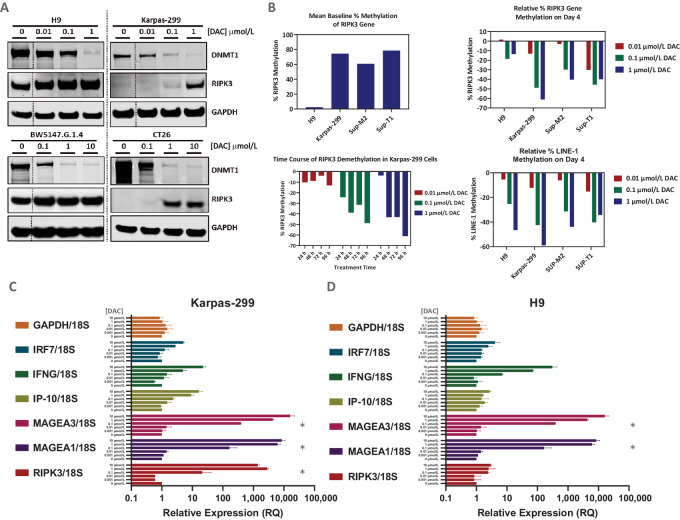
Measurement of gene expression changes in TCL cell lines after treatment with decitabine *in vitro*. **A,** Western blots of human (H9 and Karpas-299) and mouse (BW5147 and CT26) cell lysates prepared after treatment with decitabine for 4 days (human) or 2 days (mouse). Dotted lines indicate a cropped blot for presentation purposes. **B,** Bisulfite pyrosequencing data from four human TCL cell lines, showing basal (untreated) levels RIPK3 promoter methylation (top left), decitabine concentration-dependent decrease of RIPK3 methylation in all cell lines after 4 days treatment (top right), time dependent of RIPK3 promoter demethylation in Karpas-299 cells (bottom left), LINE-1 demethylation as control (at day 4, bottom right) for all four cell lines. Relative levels if cDNA expression measured by qPCR prepared from Karpas-299 (**C**) and H9 (**D**) cells treated for 4 days with a various concentration of decitabine. Each gene was normalized to *18S* housekeeping gene as a control. * Indicates an estimation of relative expression since this gene was not detected in DMSO control (DMSO control C_T_ set to 40).

Next, we showed that *RIPK3* silencing by promoter hypermethylation in human or mouse TCL cell lines could be reversed by treatment with decitabine (DAC; Fig. 2; Supplementary Fig. S4). A pair of human TCL cell lines with high (H9) and low (Karpas-299) basal levels of RIPK3 expression were treated for 4 days with decitabine, and RIPK3 levels were measured by Western blotting (Fig. 2A; Supplementary Fig. S11). Similarly, a pair of mouse cell lines with high (BW5147, TCL) and low (CT26, colon cancer) basal levels of RIPK3 expression were treated for 2 days with decitabine and levels of RIPK3 measured (Fig. 2A; Supplementary Fig. S11). Higher levels of RIPK3 were detected in both Karpas-299 and CT26 cells after treatment with decitabine, demonstrating that decitabine treatment can lead to re-expression of this key biomarker, which is often silenced in cancer cell lines (17).

To confirm that direct promoter demethylation is involved in the re-expression of RIPK3, we performed pyrosequencing analysis of the human *RIPK3* promoter region (Supplementary Fig. S4A) in TCL cell lines. Bisulfite-treated DNA samples isolated from untreated and decitabine-treated human TCL cell lines were used to assess the degree of CPG methylation of the *RIPK3* promoter (Fig. 2B; Supplementary Fig. S4B and S4C). As expected, H9 cells showed low basal methylation of the *RIPK3* promoter, whereas SUP-M2, SUP-T1, and Karpas-299 cells showed high basal methylation levels. Decitabine treatment led to time- and concentration-dependent decreases in *RIPK3* promoter methylation levels in all three cell lines, with high basal methylation (Fig. 2B; Supplementary Fig. S4B). All cell lines showed a similar degree of LINE-1 demethylation, a commonly used genome-wide demethylation marker, induced by HMA treatment (Fig. 2B; Supplementary Fig. S4C).

### HMA-induced Gene Expression Both Increases Cancer Testis Antigen Expression and Augments Genes Involved in IFN Signaling

We investigated the effects of decitabine treatment on the expression of additional genes by qPCR in two human cell lines, Karpas-299 and H9. HMA treatment has been reported to re-express certain genes that are silenced by promoter methylation, such as cancer testis antigens (CTA), in human TCL cell lines (26). We selected two CTA genes (*MAGEA1* and *MAGEA3*) along with *RIPK3* and interferon signaling genes (ISG), which are known to be upregulated by HMA, including IFNγ (*IFNG*; refs. 27, 28), IRF7, and IP-10 (29, 30). IP-10, a chemokine previously shown to be modulated by tolinapant treatment (11), increased upon decitabine treatment (Fig. 2C and D). *RIPK3* expression was upregulated in Karpas-299 cells (Fig. 2C) and to a lesser degree in H9 cells (Fig. 2D), as expected, owing to the elevated basal RIPK3 level in H9 cells. MAGEA1 and MAGEA3 expression was upregulated in both cell lines and ISGs were upregulated in both cell lines.

### IAP Antagonism and Hypomethylation Have an Antitumor Effect on TCL Cell Lines *In Vitro* with a Mechanism Involving ICD

**FIGURE 3:**
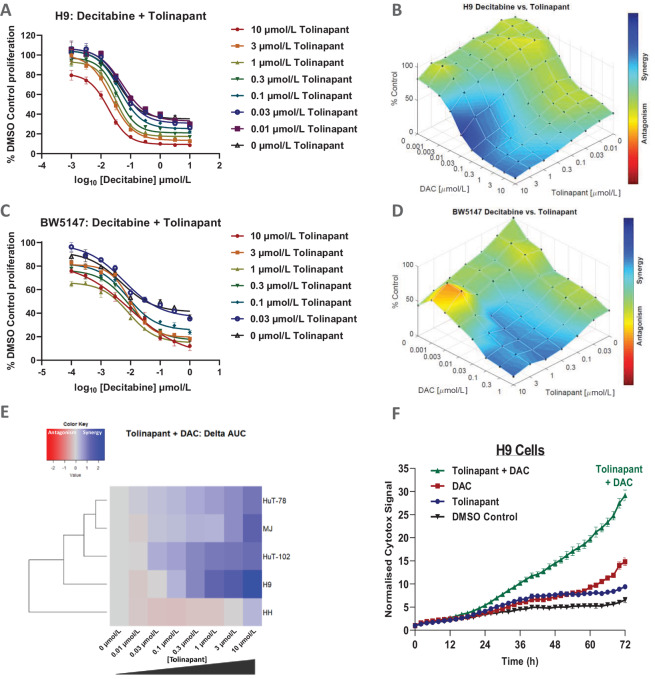
Synergistic interaction between decitabine and tolinapant in reducing viability of TCL cell lines *in vitro.* Viability data obtained using CTG in H9 cells (**A** and **B**) and in BW5147 cells (**C** and **D**) after 3 days of treatment with a combination of decitabine and tolinapant (A and C) raw data and (B and D) HSA score from ComBenefit synergy analysis. **E,** Heat map showing a change in AUC measurements obtained from 3-day CTG assays in five different human TCL cell lines, testing the combination of tolinapant and decitabine (for raw data, see Supplementary Fig. S5). **F,** Cytotox-NIR signal captured by real-time microscopy (IncuCyte), detecting membrane permeabilization after the treatment of H9 cells with 1 µmol/L tolinapant, 1 µmol/L decitabine, or a combination of both.

Combined treatment with decitabine and tolinapant led to an enhanced loss of viability in both human (Fig. 3A and B) and mouse (Fig. 3C and D) TCL cell lines. Synergy, measured by the change in area under dose–response curves (AUC) calculated for each tolinapant concentration used, was detected in a panel of five human TCL cell lines (Fig. 3E; Supplementary Fig. S5). Using real-time microscopy with Cytotox-NIR, we demonstrated that H9 cells treated with the combination underwent lytic cell death (Fig. 3F). The levels of cell staining, indicative of lytic cell death, were significantly higher than those in cells treated with either monotherapy.

**FIGURE 4:**
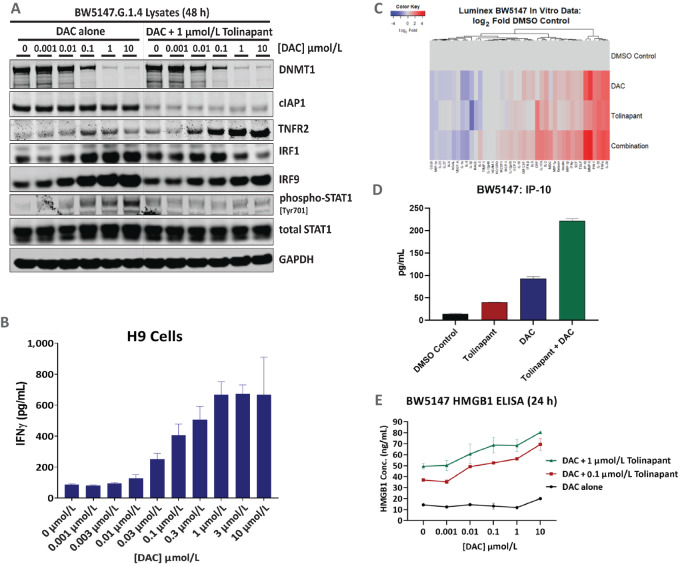
Increased levels of IFN signaling components and changes in ICD biomarkers or chemokines/cytokines on treatment of TCL cell lines with decitabine and tolinapant. **A,** Western blots of mouse BW5147 cell lysates prepared after 48 hours treatment with different concentrations of decitabine without and with 1 µmol/L tolinapant. **B,** IFNγ MSD assay of H9 cell supernatants collected after 48 hours treatment with different concentrations of decitabine. **C,** log_2_ fold increase (relative to DMSO control) in 41 chemokines/cytokines from BW5147 cell supernatants taken 48 hours after treatment with 1 µmol/L DAC, 1 µmol/L tolinapant, or a combination of both measured by Luminex assay. **D,** Data from C showing raw data for IP-10 (CXCL10) levels. **E,** HMGB1 ELISA assay of BW5147 cell supernatants collected after 24 hours treatment with different concentrations of decitabine with 0, 0.1, or 1 µmol/L tolinapant.

Elevated levels of the IFN signaling markers phospho-STAT1, IRF1, and IRF9 were detected by Western blotting in the murine TCL cell line BW5147 after treatment with decitabine alone or in combination with tolinapant (Fig. 4A). IRF1 has been reported to be a critical dual regulator of IAP antagonist-induced apoptosis and inflammatory cytokine response (31), and IRF9 forms part of the master IFN-induced regulator ISGF3 complex (32). After treatment with decitabine alone or in combination with tolinapant, an increase in TNFR2 levels was also observed. Interestingly, we previously reported that TNFR2 levels increased in human PTCL patient samples after tolinapant treatment (11). A dose-dependent increase in IFNγ was observed in H9 cell supernatants upon treatment with decitabine (Fig. 4B), consistent with the increase in *IFNG* mRNA expression (Fig. 2D). A panel of four cytokines was also elevated upon treatment of Karpas-299 cells with decitabine alone or a combination of decitabine and tolinapant (Supplementary Fig. S6).

The effects of the two agents, alone and in combination, on cytokine release from BW5147 cells were analyzed. The levels of some cytokines were increased by decitabine or tolinapant alone, and were enhanced by combination treatment (Fig. 4C). One such example is IP-10 (Fig. 4D). Tolinapant treatment led to the release of HMGB1, a damage-associated molecular pattern (DAMP) biomarker in BW5147 cells (11). This effect was enhanced in the presence of decitabine, which alone did not affect HMGB1 release (Fig. 4E).

### ICD Biomarkers are Elevated in *In Vivo* TCL Tumors After Combined Treatment with HMA and IAP Antagonist

**FIGURE 5:**
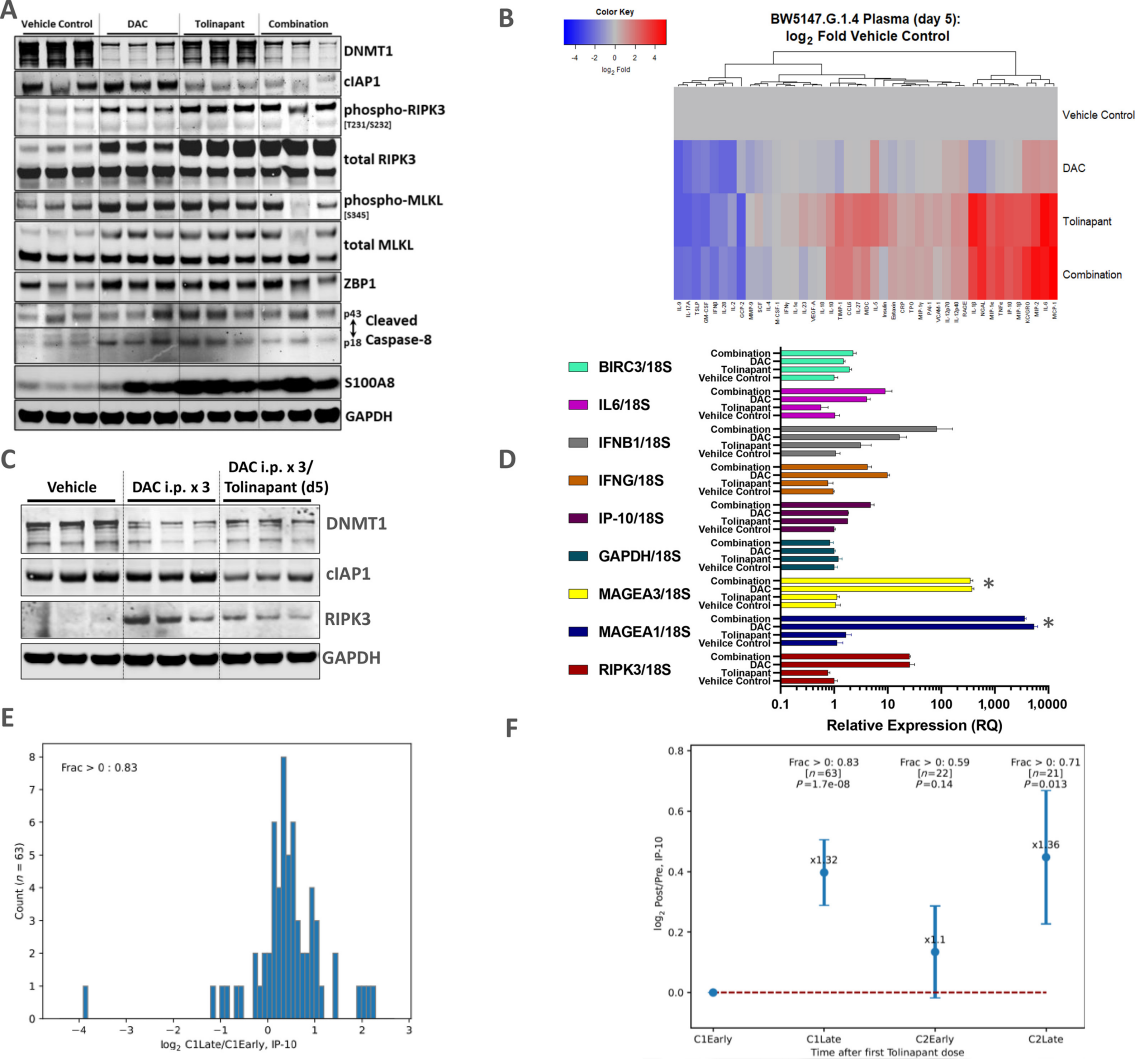
Pharmacodynamic changes in ICD biomarkers, chemokines/cytokines, and gene expression in models of TCL *in vivo* and in patients. **A,** Western blots of tumor lysates from a BW5147 tumors grown in wild-type mice. Tumor-bearing animals were treated with five daily doses of each compound alone or in combination then culled 2 hours after the final dose. **B,** Changes (log_2_ fold change relative to vehicle control) in a panel of 43 chemokine/cytokines measured by Luminex assay in plasma of the treated animals. **C,** Protein analysis by Western blots and of lysates from Karpas-299 tumors in CB17 SCID mice. Tumors were taken after three daily doses of each compound alone or in combination. **D,** Relative expression by qPCR analysis from RNA extracted from tumors taken after three daily doses of compounds as described in C. Signal for each gene was normalized to the level of *18S* housekeeping as control. * Indicates an estimation of relative expression because this gene was not detected in vehicle control (vehicle control C_T_ set to 40). **E,** Histogram showing relative change in IP-10 levels. The data are represented as log_2_ fold change in PTCL patient (*n* = 63 patients) between early times in C1 and late times in cycle C1. “Early” refers to pretreatment and 2 hours posttreatment samples; “late” refers to mostly day 2 (after the first dose), day 7, and day 8 at the end of 7 daily doses in C1. **F,** Longitudinal data showing relative plasma IP-10 levels in patients during the first two cycles. C1 (as described above) pattern was repeated during cycle 2 (C2). Cycle 2 was initiated 7 days after the last tolinapant administration. *P* values from binomial one-tailed test.

Biomarker modulation was investigated in *in vivo* pharmacodynamic studies using TCL models (Fig. 5; Supplementary Fig. S7). The tolinapant-sensitive BW5147 syngeneic model was used to test the effect of treatment with each compound alone or in combination for 5 days. At the 2-hour timepoint after the final dose, target engagement by tolinapant and decitabine was confirmed by a reduction in cIAP1 or DNMT1 levels, respectively (Fig. 5A). A clear increase in phospho-RIPK3 and phospho-MLKL levels was detected in the tumors of decitabine- or tolinapant-treated animals, suggesting necroptotic potential. There was no evidence of increased caspase-8 cleavage, which suggested that apoptosis was inhibited. The compound treatments also led to an increase in Z-DNA binding protein 1 (ZBP1), a cytoplasmic DNA sensor upregulated by IFN signaling, and a more pronounced increase in the receptor for advanced glycation end products (RAGE) ligand, S100A8. The levels of chemokines and cytokines in the plasma samples obtained from the same pharmacodynamic study were analyzed using the Luminex assay (Fig. 5B). IP-10 was elevated in the plasma of animals treated with the combination of compounds for 5 days (Fig. 5B), as was observed for KC-GRO, MIP-2, MCP-1 (Supplementary Fig. S7A), and HMGB1 (Supplementary Fig. S7B).

Decitabine-induced re-expression of RIPK3 *in vivo* was demonstrated in Karpas-299 xenografts. After 3 days of treatment, RIPK3 protein and mRNA levels increased (Fig. 5C and D). Furthermore, *MAGEA1* and *MAGEA3* CTA expression increased after decitabine treatment, like that of other chemokine and cytokine genes (*IP-10*, *IFNG, IL6,* and *IFNB1*). The increase in plasma IP-10 protein levels mirrored the increase in tumor IP-10 mRNA levels observed by qPCR (Supplementary Fig. S7C).

### IP-10 is Elevated in Plasma Samples from Patients with PTCL After Dosing with Tolinapant

We analyzed plasma samples from 63 patients with PTCL after tolinapant treatment to determine systemic levels of IP-10 using the Luminex assay (Fig. 5E and F). Between the early times in C1 and the late times in C1, an increase in plasma IP-10 was detected (Fig. 5E). The data in Fig. 5F show the longitudinal changes in the average log_2_ fold changes relative to the cycle 1 day1 early samples. These data confirm and expand our previous finding (11) that IP-10 levels are increased in patient plasma samples after tolinapant dosing.

### Engaging Necroptosis Plays a Key Role in Outcome of the Combination Therapy

**FIGURE 6:**
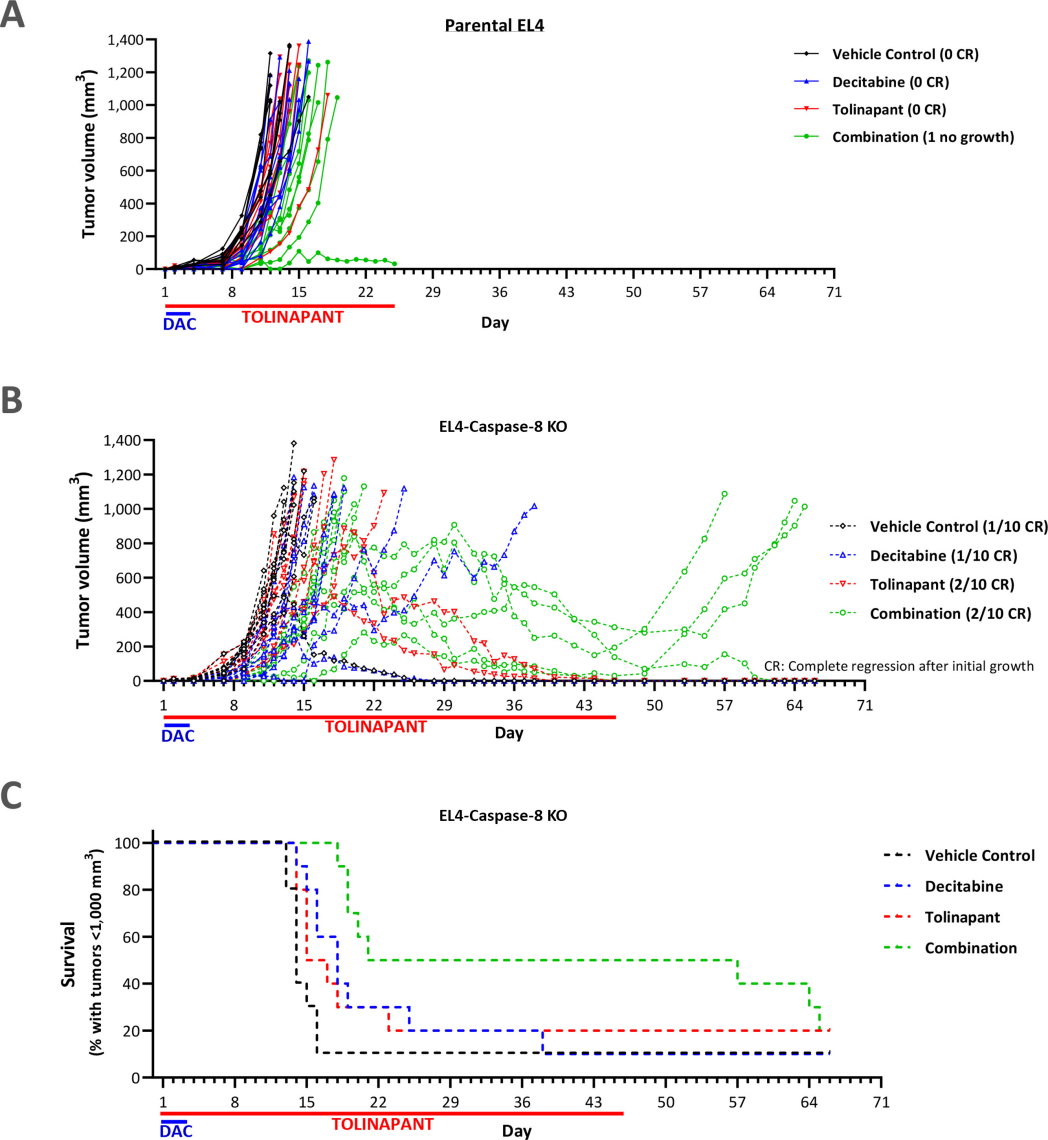
Dosing with a combination of tolinapant plus decitabine drives increased efficacy in necroptosis-model of TCL (EL4-C8KO model). Volumes of EL4-PAR (**A**) and C8KO (**B**) EL4 tumors in wild-type mice treated with decitabine (0.3 mg/kg i.p., every day × 3 doses) and tolinapant dosing (25 mg/kg orally, every day × up to 46 doses) as single agents or in combination. **C,** Kaplan–Meier plot showing survival (tumors reaching 1,000 mm^3^ as endpoint) of mice bearing EL4-C8KO (C8KO) tumors.

The syngeneic mouse TCL model EL4 showed limited sensitivity to either decitabine or tolinapant as a single agent (Fig. 6A; Supplementary Fig. S8A). Combination treatment reduced tumor growth, but nine of 10 tumors still reached the endpoint of 1,000 mm^3^ by day 19. EL4-C8KO tumors showed moderately enhanced growth inhibition by either the agent alone or in combination (Fig. 6B; Supplementary Fig. S8A). In contrast to the EL4-PAR tumors, half of the combination-treated EL4-C8KO tumor-bearing mice did not reach the endpoint by day 22, and their tumors slowly regressed over the subsequent three weeks of treatment. Upon cessation of tolinapant treatment, three out of five tumors regrew, implying that tolinapant continued to have an active role in tumor control. Nonetheless, complete regression was observed in the remaining two tumors. These data are summarized as survival proportions for each group in Fig. 6C; Supplementary Fig. S8B and S8C. Notably, 3 days of decitabine treatment at a low dose at the start of the study had a profound effect on the control of the combination-treated tumors, with a median survival time of 39 days, which was significantly longer than the 14 days observed in the vehicle group (Supplementary Fig. S8C). The body weight data suggested that the combination was tolerated (Supplementary Fig. S8D). The difference between the parental and C8 KO EL4 models suggests that when apoptosis is blocked in TCL, treatment with tolinapant plus HMA can lead to a switch to necroptosis, a form of ICD.

EL4-PAR and EL4-C8KO syngeneic model tumors and plasma samples from a pharamcodynamic study were investigated for evidence that the combination of tolinapant with decitabine acts to enhance ICD *in vivo* (Supplementary Fig. S9). IHC analysis showed that increased CD8^+^ T-cell infiltration could be measured in EL4-C8KO tumors, especially at day 12 (Supplementary Fig. S9A), and this was supported by increased levels of *CD8A* and *CD8B1* being measured in two of three tumors by qPCR (Supplementary Fig. S9C). Plasma sample analysis demonstrated a significant effect of the combination of agents on levels of IP-10 in both EL4-PAR and EL4-C8KO tumor-bearing mice at day 5 (Supplementary Fig. S9B) demonstrating that T cells can be recruited in both models, but that only in the model with increased levels of necroptosis is there a dramatic effect on tumor regression (Fig. 6).

## Discussion

Multiple cell death–inducing pathways are involved in the intervention of therapeutic molecules that target various cancers. IAP antagonists were initially developed to induce apoptosis. Recently, we and others have shown that some tumor cells undergo necroptosis following treatment with tolinapant, an antagonist of cIAP1/2 and XIAP (11). In contrast to apoptosis, necroptosis is strongly immunogenic (33), and its successful engagement could lead to robust and durable antitumor immune effects. In addition, tolinapant has recently demonstrated single-agent activity in relapsed/refractory PTCL and CTCL clinical trials (12, 13). This led us to demonstrate an immune-based mechanism of action of the compound in preclinical TCL models and in samples from patients treated with tolinapant (11). Understanding that tolinapant treatment can drive ICD, particularly necroptosis, in TCL models has opened new avenues for improving patient outcomes through combination options. Progress in the development of alternative IAP antagonists in combination clinical trials in several cancer types (34) has prompted us to test additional combination options for tolinapant preclinically (35–38) in TCL. In this study, we sought to provide a rationale for a novel combination therapy of HMA and IAP antagonism in TCL.

By systematically using CRISPR to target the key necroptosis signaling components RIPK3, MLKL, and caspase-8, we confirmed that tolinapant drives lytic cell death via necroptosis in TCL cell lines. We showed the requirement for RIPK3 and MLKL expression (39–41), and that necroptosis could be induced under conditions in which caspase-8 is inhibited, as has been shown for other IAP antagonists in different settings (38, 42, 43). We also confirmed the release of proinflammatory markers by measuring cytokine and chemokine production during necroptosis induction (40).

Similarly, epigenetic alterations have been reported to play an important role in the pathogenesis and development of some subtypes of PTCL, and the regulatory approval of romidepsin, a histone deacetylase inhibitor, demonstrates that PTCL is sensitive to epigenetic intervention (44). One of the most prevalent epigenetic alterations is DNA methylation (45), and early clinical trials have demonstrated the activity of HMA as a single agent in PTCL (20, 21). Epigenetic silencing is a mechanism by which cancer cells evade necroptosis (17). Therefore, we investigated the effects of decitabine, an HMA, on tolinapant-induced necroptosis (11) in TCL, and the immunomodulatory potential of this agent.

A recent CRISPR/Cas9 whole-genome screen investigating necroptosis resistance in mouse fibroblast cells identified necrosome components (including RIPK1, RIPK3, and MLKL) as well as regulators and mediators of necroptosis (46), indicating their importance in necroptosis. The expression of key components of the necrosome, such as RIPK3, is epigenetically regulated (17). We confirmed that RIPK3 was silenced by promoter methylation in a panel of human TCL cell lines and that this methylation could be reversed by treatment with decitabine, providing a route of sensitization to an IAP antagonist in combination (38, 47). We demonstrated that, in the TCL cell lines tested, a synergistic loss of viability could be achieved by the combination of decitabine and tolinapant *in vitro*. In addition to direct re-expression of RIPK3, decitabine has multiple effects that are expected to enhance immune stimulation. Decitabine also upregulates MLKL, another key component of the necrosome, possibly via increased IFNγ signaling (27, 28). The increased levels of known tumor antigens (e.g., CTAs) invoked by HMA-driven re-expression can further increase the immune recognition of tumors (48). Our studies confirmed these findings in TCL cell lines, strongly suggesting that decitabine can prime tumor cells toward immune-mediated killing by the addition of tolinapant (11).

We then continued the pharmacologic exploration *in vivo.* Necroptosis has been demonstrated in a sensitive BW5147 syngeneic model with both single agents and in combination (11). This suggested that decitabine itself has the potential to drive necroptosis in our TCL model. We showed that HMA-driven enhancement of RIPK3 and CTAs, along with increased chemokine and cytokine levels, can be measured in preclinical models of TCL. We focused on IP-10, a key chemokine that attracts CXCR3-positive T cells, and found that levels were consistently increased in our preclinical TCL models*.* This prompted us to test whether elevated levels of this chemokine could be detected in the plasma samples of patients with PTCL after tolinapant treatment. Increased plasma levels of IP-10 after tolinapant dosing were measured, providing further evidence for the immunomodulatory modality of IAP antagonism in T-cell recruitment and confirming the relevance of our preclinical models and mode-of-action studies in the clinical arena.

To further profile the *in vivo* response to tolinapant and decitabine combination treatment, we employed the syngeneic EL4-C8KO model, which was established as a necroptosis model owing to the role of caspase-8 in preventing necrosome formation (8). A short, low-dose epigenetic priming regimen with decitabine to initiate the study allowed tolinapant to exert a robust antitumor effect in this model and expand its immunogenic potential. The EL4-PAR syngeneic tumor model is poorly immunogenic (49). Therefore, it was notable that deletion of a single gene, caspase-8, in the necrosome could sensitize cells to decitabine and tolinapant. Both agents showed significant but limited single-agent activity against C8-KO EL4 tumors, and neither single-agent treatment exerted significant antitumor activity to fully reduce tumor growth. Remarkably, however, the combination of both treatments led to a significantly longer response duration. Any tumor that initially grew eventually shrank with continued tolinapant treatment. The regrowth of regressing tumors upon treatment withdrawal suggests that tolinapant continued to play an active role in tumor control. Overall, the study results suggest that both initial necroptosis and sustained immune engagement are key to tumor control.

We believe that the combination of HMA and IAP antagonists enhances ICD (Supplementary Fig. S10) and induces a robust immune response against a tumor with an intact necrosome. Samples from the Ascertain-P clinical study (NCT05403450), in which PTCL patients are being treated with tolinapant and ASTX727 (oral decitabine and cedazauridine), will enable additional exploration of the tumor-directed immunomodulatory response described in this article.

## Supplementary Material

Table S1 Table S1. Details of primary antibodies used in Western blots in this study (Figures 1,2,4,6 & S1).

Table S2 Table S2. Details of TaqMan primers used in qPCR assays in this study (Figures 2,5 & S9).

Table S3 Table S3. Generation of CRISPR Knockout (KO) and CRISPR Activation (CRISPRa) Cell Line Clones

Figure S1 Figure S1. Additional Western blots showing different CRISPR clones generated from each construct. (Refers to Figure 1)

Figure S2 Figure S2. Additional viability and cytokine data from each TCL CRISPR clone experiment. (Refers to Figure 1)

Figure S3 Figure S3. Additional EL4-C8KO Western blot and lytic cell death data. (Refers to Figure1)

Figure S4 Figure S4. Changes in DNA methylation levels after decitabine (DAC) treatment of human TCL cell lines. (Refers to Figure 2)

Figure S5 Figure S5. Combination viability assay raw data. (Refers to Figure 3)

Figure S6 Figure S6. Additional Karpas-299 cytokine data. (Refers to Figure 4)

Figure S7 Figure S7. Additional in vivo PD data. (Refers to Figure 5)

Figure S8 Figure S8. Additional EL4 & EL4-C8KO model in vivo efficacy data. (Refers to Figure 6)

Figure S9 Figure S9. Analysis of tumor and plasma samples from EL4-Parental and EL4-C8KO syngeneic model PD studies (Refers to Figure 6)

Figure S10 Figure S10. Impact of tolinapant and decitabine on induction of immunogenic cell death in TCL. (Refers to Discussion)

Figure S11 Uncropped Western blots from Figure 2A
