# Supplementary material for: Epigenetic Priming by Hypomethylation Enhances the Immunogenic Potential of Tolinapant in T-cell Lymphoma
Source: Cancer Res Commun. 2024 Jun 6;4(6):1441–53. doi: 10.1158/2767-9764.CRC-23-0415 (PMC11155518; doi:10.1158/2767-9764.CRC-23-0415)
Supplement: Table S1 — Details of primary antibodies used in Western blots in this study (Figures 1,2,4,6 & S1). [file crc-23-0415-s01.docx]

**Table S1**. Details of primary antibodies used in Western blots in this study (Figures 1,2,4,6 & S1).

| **Antibody** | **Supplier** | **Cat. No. &**  **RRID** | **Dilution** |
| --- | --- | --- | --- |
| Mouse cIAP1 | Enzo Life Sciences (New York, USA) | ALX-803-335  RRID:AB_2227905 | 1:1000 |
| Human cIAP1 | R&D Systems (Abingdon, UK) | AF8181  RRID:AB_2259001 | 1:1000 |
| Human/Mouse DNMT1 | Cell Signaling Technology (Hitchin, UK) | #5032  RRID:AB_10548197 | 1:1000 |
| Human phospho-RIPK3 [S227] | Cell Signaling Technology (Hitchin, UK) | #93654  RRID:AB_ 2800206 | 1:1000 |
| Mouse phospho-RIPK3 [T231/S232]) | Cell Signaling Technology (Hitchin, UK) | #91702  RRID:AB_2937060 | 1:1000 |
| Human RIPK3 | Cell Signaling Technology (Hitchin, UK) | #13526  RRID:AB_2687467 | 1:1000 |
| Mouse RIPK3 | Cell Signaling Technology (Hitchin, UK) | #95702  RRID:AB_2721823 | 1:1000 |
| Human phospho-MLKL [S358] | Cell Signaling Technology (Hitchin, UK) | #91689  RRID:AB_2895044 | 1:1000 |
| Mouse phospho-MLKL [S345] | Cell Signaling Technology (Hitchin, UK) | #37333  RRID:AB_2799112 | 1:1000 |
| Human/Mouse MLKL | Abcam (Cambridge, UK) | ab243142  RRID:AB_3083655 | 1:1000 |
| Mouse Caspase-8 | Cell Signaling Technology (Hitchin, UK) | #4927  RRID:AB_2068301 | 1:1000 |
| Mouse Cleaved Caspase-8 [Asp387] | Cell Signaling Technology (Hitchin, UK) | #8592  RRID:AB_10891784 | 1:1000 |
| Human/Mouse Cleaved Caspase-3 [Asp175] | Cell Signaling Technology (Hitchin, UK) | #9664  RRID:AB_ 2070042 | 1:500 |
| Human/Mouse phospho-H2A.X [S139] | Cell Signaling Technology (Hitchin, UK) | #2577  RRID:AB_2118010 | 1:1000 |
| Mouse ZBP1 | AdipoGen Life Sciences (Füllinsdorf, Switzerland) | AG-20B-0010  RRID:AB_2490191 | 1:1000 |
| Human/Mouse TNFR2 | Cell Signaling Technology (Hitchin, UK) | #3727  RRID:AB_659912 | 1:1000 |
| Human/Mouse IRF1 | Cell Signaling Technology (Hitchin, UK) | #8478  RRID:AB_10949108 | 1:1000 |
| Mouse IRF9 | Cell Signaling Technology (Hitchin, UK) | #28845  RRID:AB_2798964 | 1:1000 |
| Human/Mouse Phospho-STAT1 [Y701] | Cell Signaling Technology (Hitchin, UK) | #9167  RRID:AB_561284 | 1:1000 |
| Human/Mouse STAT1 | Cell Signaling Technology (Hitchin, UK) | #14994  RRID:AB_2737027 | 1:1000 |
| Mouse S100A8 | R&D Systems (Abingdon, UK) | AF3059  RRID:AB_2184254 | 1:1000 |
