# Supplementary material for: Epigenetic Priming by Hypomethylation Enhances the Immunogenic Potential of Tolinapant in T-cell Lymphoma
Source: Cancer Res Commun. 2024 Jun 6;4(6):1441–53. doi: 10.1158/2767-9764.CRC-23-0415 (PMC11155518; doi:10.1158/2767-9764.CRC-23-0415)
Supplement: Table S2 — Details of TaqMan primers used in qPCR assays in this study (Figures 2,5 & S9). [file crc-23-0415-s02.docx]

**Table S2**. Details of TaqMan primers used in qPCR assays in this study (Figures 2,5 & S9).

| **Species** | **Gene** | **Reference Number** | **Fluorochrome** | **Cat. No. (Thermo Fisher)** |
| --- | --- | --- | --- | --- |
| Human | *RIPK3* | Hs00179132_m1 | FAM-MGB | 4331182 |
| Human | *MAGEA1* | Hs00607097_m1 | FAM-MGB | 4331182 |
| Human | *MAGEA3* | Hs03985994_uH | FAM-MGB | 4331182 |
| Human | *IFNG* | Hs00989291_m1 | FAM-MGB | 4331182 |
| Human | *IFNB1* | Hs01077958_s1 | FAM-MGB | 4331182 |
| Human | *IRF7* | Hs01014809_g1 | FAM-MGB | 4331182 |
| Human | *IL-6* | Hs00985639_m1 | FAM-MGB | 4331182 |
| Human | *BIRC3* | Hs00985031_g1 | FAM-MGB | 4331182 |
| Human | IP-10 | Hs00171042_m1 | FAM-MGB | 4331182 |
| Human | *GAPDH* | Hs99999905_m1 | FAM-MGB | 4331182 |
| Human | *18S* | Hs99999901_s1 | VIC-MGB | 4448484 |
| Mouse | *CD8A* | Mm01182107_g1 | FAM-MGB | 4331182 |
| Mouse | *CD8B1* | Mm00438116_m1 | FAM-MGB | 4331182 |
| Mouse | *GAPDH* | Mm99999915_g1 | FAM-MGB | 4331182 |
| Mouse | *18S* | Mm03928990_g1 | VIC-MGB | 4448485 |
