# Supplementary material for: Epigenetic Priming by Hypomethylation Enhances the Immunogenic Potential of Tolinapant in T-cell Lymphoma
Source: Cancer Res Commun. 2024 Jun 6;4(6):1441–53. doi: 10.1158/2767-9764.CRC-23-0415 (PMC11155518; doi:10.1158/2767-9764.CRC-23-0415)
Supplement: Table S3 — Generation of CRISPR Knockout (KO) and CRISPR Activation (CRISPRa) Cell Line Clones [file crc-23-0415-s03.docx]

**Table S3. Generation of CRISPR Knockout (KO) and CRISPR Activation (CRISPRa) Cell Line Clones**

| 1. **EL4-C8KO (Horizon, Cambridge, UK)**   KO of the 4 alleles of mouse *caspase-8* (*Casp8*: ENSMUSG00000026029) on chromosome 1 (58,795,374-58,847,503 forward strand) was achieved using the gRNA 5’-AATGGAGAAGAGGACCATGC-3’ targeting exon 4. Gene edit outcomes were evaluated by PCR (717-bp band using 5’-CCTCAGGCTGGTCCATAGCT-3’ forward and 5’-TGTTTCCATGGGGCTTTCAGAG-3’ reverse primers) and sequencing around gRNA targeting site (using sequencing primer 5’-CCTCAGGCTGGTCCATAGCT-3’). Two positive clones were confirmed to have caspase-8 KO on all alleles (*Casp8*^(-/-/-/-)^). |
| --- |
| 1. **BW5147-MLKLKO (Horizon, Cambridge, UK)**   KO of the 2 alleles of mouse *Mlkl* (*Mlkl*: ENSMUSG00000012519) on chromosome 8 (111,311,797-111,338,177 reverse strand) was achieved using the gRNA 5’-GTCTTCAGTTTGGTCCACGG-3’ targeting exon 4. Gene edit outcomes were evaluated by PCR (455-bp band using 5’-CGCAAAGCTTCACTGCCAAC-3’ forward and 5’-TGACACCCTCACCCCCATTT-3’ reverse primers) and sequencing around gRNA targeting site (using sequencing primer 5’-TGACACCCTCACCCCCATTT-3’). Two positive clones were confirmed to have Mlkl KO on both alleles (*Mlkl*^(-/-)^). |
| 1. **BW5147-RIPK3KO (Horizon, Cambridge, UK)**   KO of the 2 alleles of mouse *Ripk3* (*Ripk3*: ENSMUSG00000022221) on chromosome 14 (55,784,995-55,788,865 reverse strand) was achieved using the gRNA 5’- GCGGAGGGTTCAAGCTGTGT -3’ targeting exon 3. Gene edit outcomes were evaluated by PCR (767-bp band using 5’- TTCCTCTGGTGAGCCGTGAA-3’ forward and 5’- CTGAGTTGCTGATGGGCAGG-3’ reverse primers) and sequencing around gRNA targeting site (using sequencing primer 5’- TTCCTCTGGTGAGCCGTGAA-3’). Two positive clones were confirmed to have Ripk3 KO on both alleles (*Ripk3*^(-/-)^). |
| **iv) Karpas-299-RIPK3 (CRISPRa)**  Up-regulation of silent endogenous RIPK3 gene in Karpas299 cells was achieved using CRISPRa. Cassette of dCas9-VPR constitutive expression (hEF1α-Blast-dCas9-VPR) was introduced by lentiviral particle transduction (Horizon, cat. no. VCAS11922) of Karpass299 cells at MOI 1.0 (1 TU to 1 cell) with 5 μg/ml polybrene (Merck, cat. no. H9268). Selection was performed by adding 15 μg/ml blasticidin S (Thermo Fisher, cat. no. A1113903) for 15 days. Single cell clones were sorted using a BD FACS Melody. Expression of dCas9-VPR was detected by Western blotting with anti-Cas9 antibody (Abcam, cat. no. ab191468).  A clone with high dCas9-VPR expression was used for RIPK3 up-regulation by lentiviral transduction using the expression cassette (U6-sgRNA-mCMV-Puro) containing gRNA targeting RIPK3 gene promoter region (Horizon, cat. no. VSGH11888-247220546, VSGH11888-247220547, VSGH11888-247220548 and VSGH11888-247220549). Non-targeting control gRNA (Horizon, cat. no. VSGC11311) was used for the control cell line. Cells were selected with 2 μg/ml puromycin dihydrochloride (Thermo Fisher, cat. no. A1113803). Single cells were sorted by BD FACS Melody and expression of RIPK3 was detected by Western blotting using RIPK3 antibody (Table S1). Karpas299 cells with no RIPK3 expression (transduced with non-targeting control gRNA), low expression (transduced with gRNA 461), intermediate (transduced with gRNA 462) and high expression (transduced with gRNA 462) were used for further experiments. |
