# Supplementary material for: Epigenetic Priming by Hypomethylation Enhances the Immunogenic Potential of Tolinapant in T-cell Lymphoma
Source: Cancer Res Commun. 2024 Jun 6;4(6):1441–53. doi: 10.1158/2767-9764.CRC-23-0415 (PMC11155518; doi:10.1158/2767-9764.CRC-23-0415)
Supplement: Figure S1 — Additional Western blots showing different CRISPR clones generated from each construct. (Refers to Figure 1) [file crc-23-0415-s04.pptx]

## Slide 1
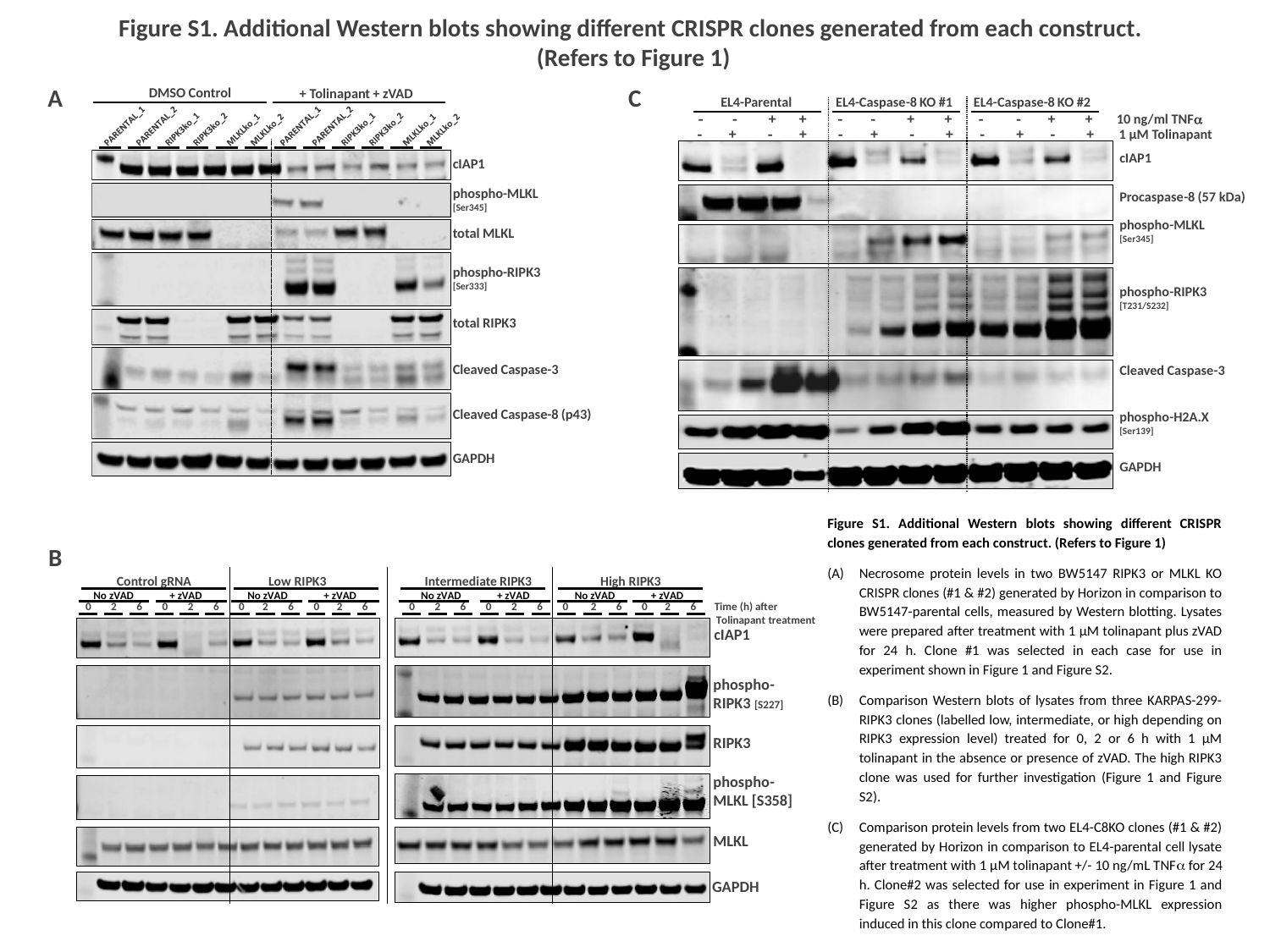

Figure S1. Additional Western blots showing different CRISPR clones generated from each construct.
(Refers to Figure 1)
A
C
Figure S1. Additional Western blots showing different CRISPR clones generated from each construct. (Refers to Figure 1)
Necrosome protein levels in two BW5147 RIPK3 or MLKL KO CRISPR clones (#1 & #2) generated by Horizon in comparison to BW5147-parental cells, measured by Western blotting. Lysates were prepared after treatment with 1 µM tolinapant plus zVAD for 24 h. Clone #1 was selected in each case for use in experiment shown in Figure 1 and Figure S2.
Comparison Western blots of lysates from three KARPAS-299-RIPK3 clones (labelled low, intermediate, or high depending on RIPK3 expression level) treated for 0, 2 or 6 h with 1 µM tolinapant in the absence or presence of zVAD. The high RIPK3 clone was used for further investigation (Figure 1 and Figure S2).
Comparison protein levels from two EL4-C8KO clones (#1 & #2) generated by Horizon in comparison to EL4-parental cell lysate after treatment with 1 µM tolinapant +/- 10 ng/mL TNFa for 24 h. Clone#2 was selected for use in experiment in Figure 1 and Figure S2 as there was higher phospho-MLKL expression induced in this clone compared to Clone#1.
B
