# Supplementary material for: Epigenetic Priming by Hypomethylation Enhances the Immunogenic Potential of Tolinapant in T-cell Lymphoma
Source: Cancer Res Commun. 2024 Jun 6;4(6):1441–53. doi: 10.1158/2767-9764.CRC-23-0415 (PMC11155518; doi:10.1158/2767-9764.CRC-23-0415)
Supplement: Figure S2 — Additional viability and cytokine data from each TCL CRISPR clone experiment. (Refers to Figure 1) [file crc-23-0415-s05.pptx]

## Slide 1
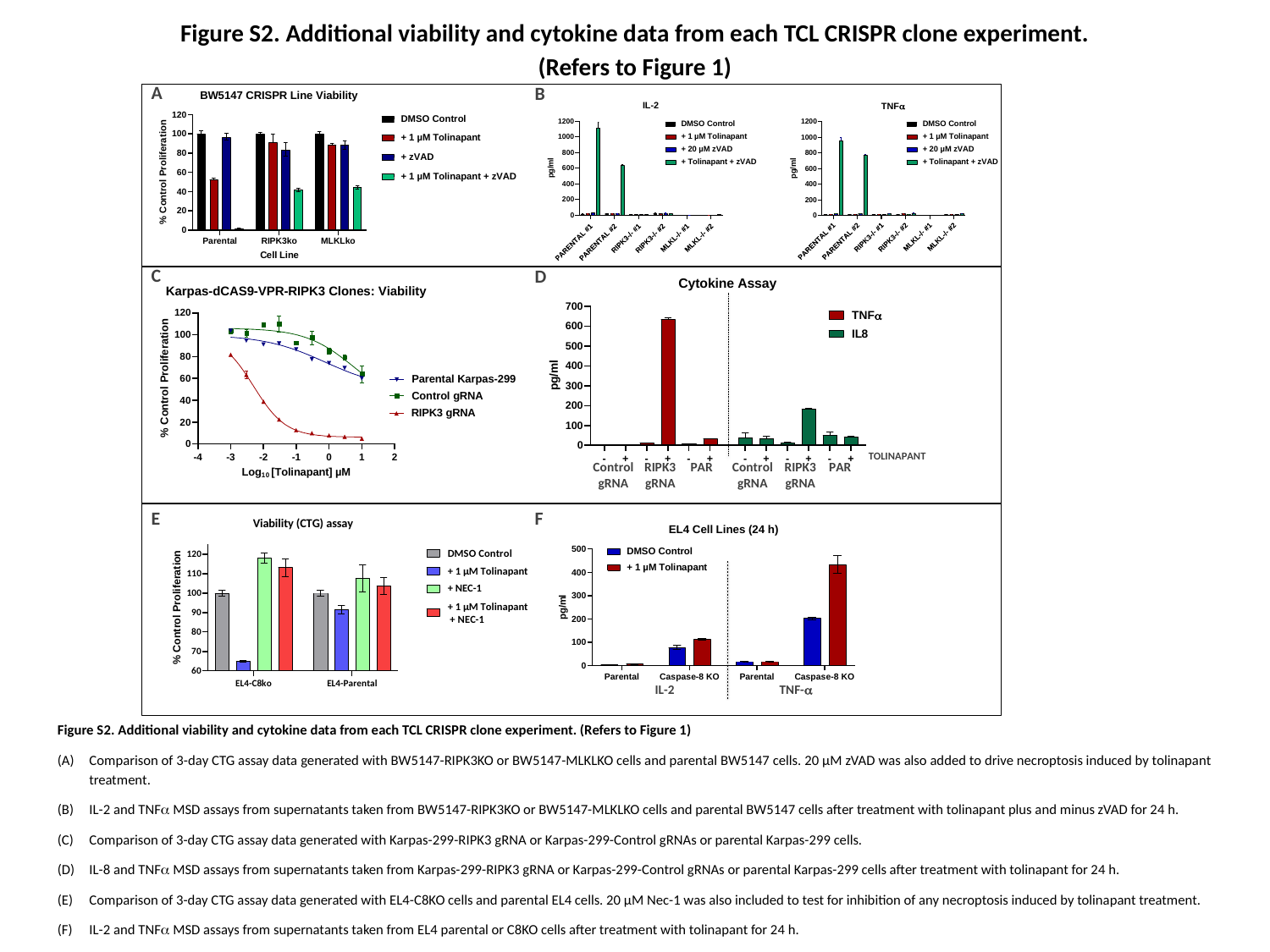

Figure S2. Additional viability and cytokine data from each TCL CRISPR clone experiment.
(Refers to Figure 1)
Figure S2. Additional viability and cytokine data from each TCL CRISPR clone experiment. (Refers to Figure 1)
Comparison of 3-day CTG assay data generated with BW5147-RIPK3KO or BW5147-MLKLKO cells and parental BW5147 cells. 20 µM zVAD was also added to drive necroptosis induced by tolinapant treatment.
IL-2 and TNFa MSD assays from supernatants taken from BW5147-RIPK3KO or BW5147-MLKLKO cells and parental BW5147 cells after treatment with tolinapant plus and minus zVAD for 24 h.
Comparison of 3-day CTG assay data generated with Karpas-299-RIPK3 gRNA or Karpas-299-Control gRNAs or parental Karpas-299 cells.
IL-8 and TNFa MSD assays from supernatants taken from Karpas-299-RIPK3 gRNA or Karpas-299-Control gRNAs or parental Karpas-299 cells after treatment with tolinapant for 24 h.
Comparison of 3-day CTG assay data generated with EL4-C8KO cells and parental EL4 cells. 20 µM Nec-1 was also included to test for inhibition of any necroptosis induced by tolinapant treatment.
IL-2 and TNFa MSD assays from supernatants taken from EL4 parental or C8KO cells after treatment with tolinapant for 24 h.
