# Supplementary material for: Epigenetic Priming by Hypomethylation Enhances the Immunogenic Potential of Tolinapant in T-cell Lymphoma
Source: Cancer Res Commun. 2024 Jun 6;4(6):1441–53. doi: 10.1158/2767-9764.CRC-23-0415 (PMC11155518; doi:10.1158/2767-9764.CRC-23-0415)
Supplement: Figure S3 — Additional EL4-C8KO Western blot and lytic cell death data. (Refers to Figure1) [file crc-23-0415-s06.pptx]

## Slide 1
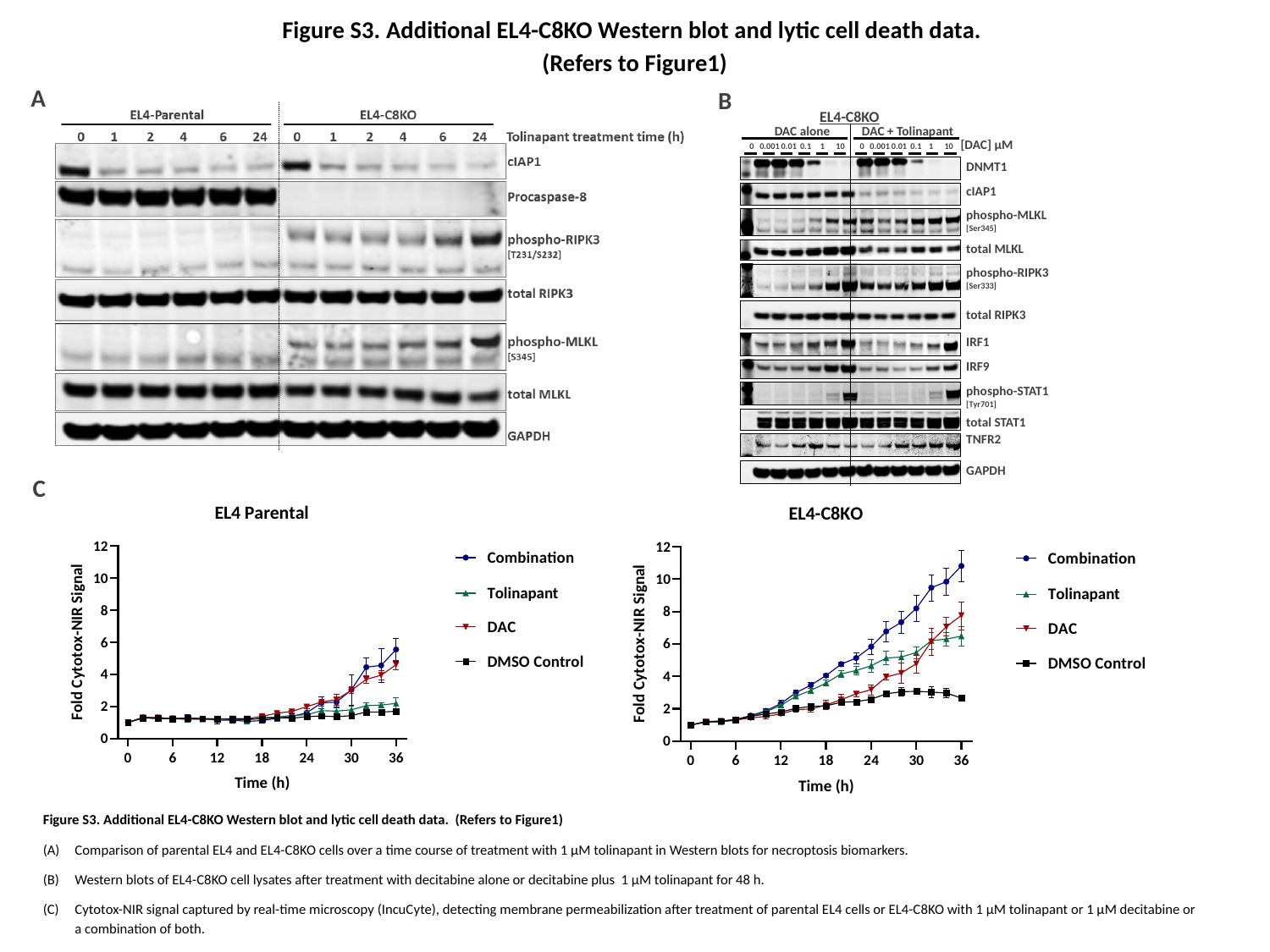

# Figure S3. Additional EL4-C8KO Western blot and lytic cell death data. (Refers to Figure1)
A
B
C
Figure S3. Additional EL4-C8KO Western blot and lytic cell death data. (Refers to Figure1)
Comparison of parental EL4 and EL4-C8KO cells over a time course of treatment with 1 µM tolinapant in Western blots for necroptosis biomarkers.
Western blots of EL4-C8KO cell lysates after treatment with decitabine alone or decitabine plus 1 µM tolinapant for 48 h.
Cytotox-NIR signal captured by real-time microscopy (IncuCyte), detecting membrane permeabilization after treatment of parental EL4 cells or EL4-C8KO with 1 µM tolinapant or 1 µM decitabine or a combination of both.
