# Supplementary material for: Epigenetic Priming by Hypomethylation Enhances the Immunogenic Potential of Tolinapant in T-cell Lymphoma
Source: Cancer Res Commun. 2024 Jun 6;4(6):1441–53. doi: 10.1158/2767-9764.CRC-23-0415 (PMC11155518; doi:10.1158/2767-9764.CRC-23-0415)
Supplement: Figure S4 — Changes in DNA methylation levels after decitabine (DAC) treatment of human TCL cell lines. (Refers to Figure 2) [file crc-23-0415-s07.pptx]

## Slide 1
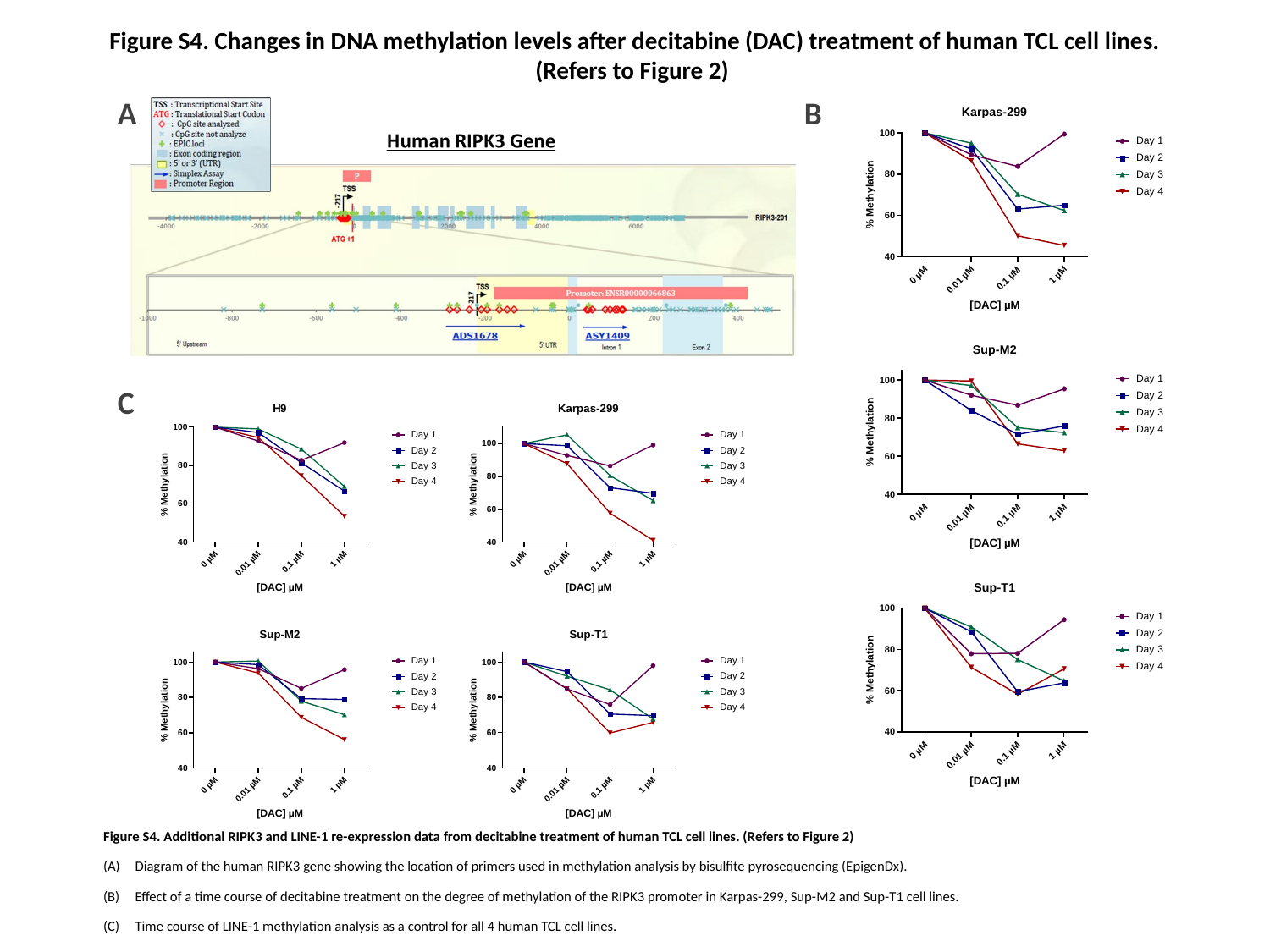

Figure S4. Changes in DNA methylation levels after decitabine (DAC) treatment of human TCL cell lines.
(Refers to Figure 2)
A
B
C
Figure S4. Additional RIPK3 and LINE-1 re-expression data from decitabine treatment of human TCL cell lines. (Refers to Figure 2)
Diagram of the human RIPK3 gene showing the location of primers used in methylation analysis by bisulfite pyrosequencing (EpigenDx).
Effect of a time course of decitabine treatment on the degree of methylation of the RIPK3 promoter in Karpas-299, Sup-M2 and Sup-T1 cell lines.
Time course of LINE-1 methylation analysis as a control for all 4 human TCL cell lines.
