# Supplementary material for: Epigenetic Priming by Hypomethylation Enhances the Immunogenic Potential of Tolinapant in T-cell Lymphoma
Source: Cancer Res Commun. 2024 Jun 6;4(6):1441–53. doi: 10.1158/2767-9764.CRC-23-0415 (PMC11155518; doi:10.1158/2767-9764.CRC-23-0415)
Supplement: Figure S5 — Combination viability assay raw data. (Refers to Figure 3) [file crc-23-0415-s08.pptx]

## Slide 1
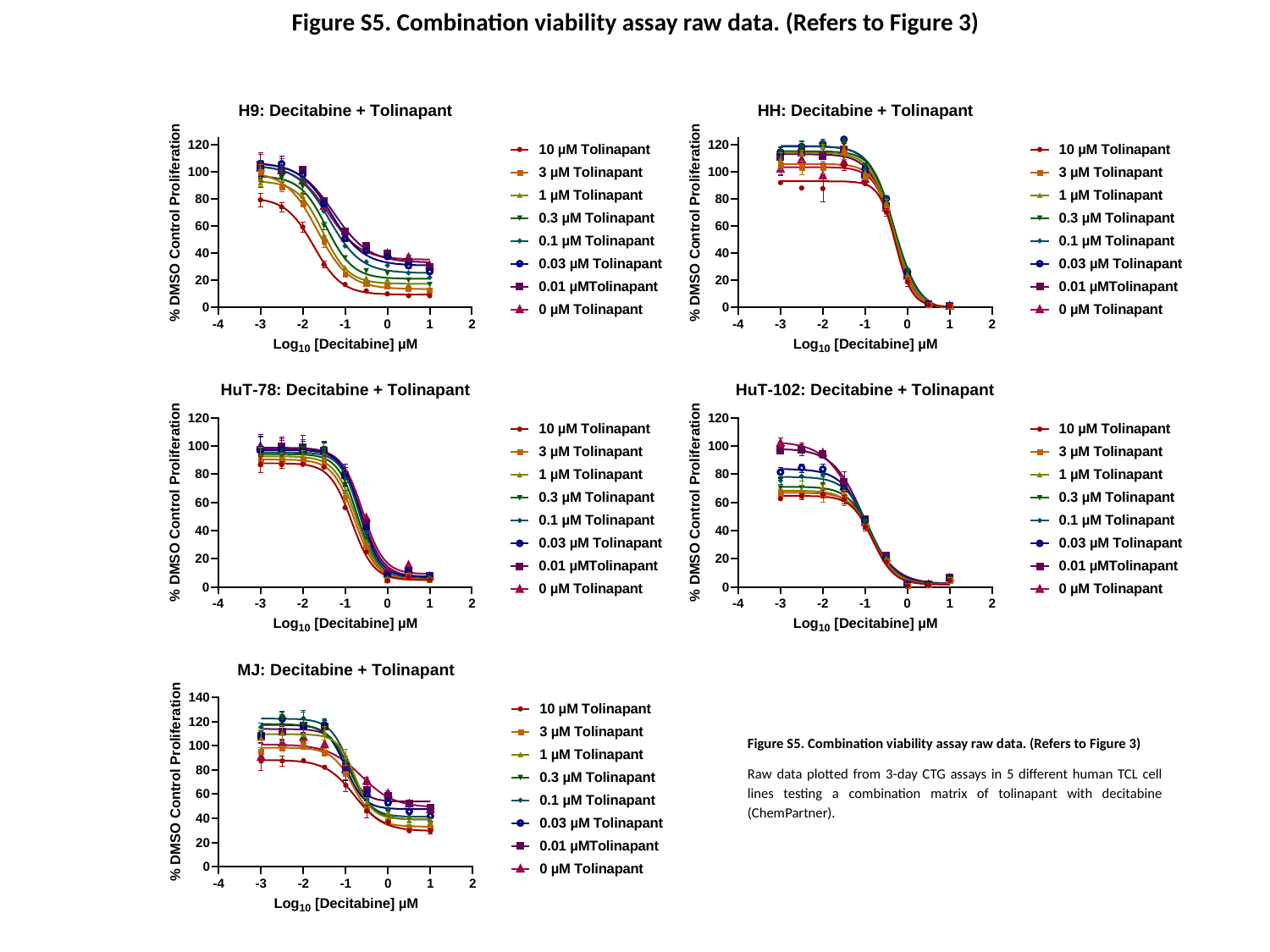

Figure S5. Combination viability assay raw data. (Refers to Figure 3)
Figure S5. Combination viability assay raw data. (Refers to Figure 3)
Raw data plotted from 3-day CTG assays in 5 different human TCL cell lines testing a combination matrix of tolinapant with decitabine (ChemPartner).
