# Supplementary material for: Epigenetic Priming by Hypomethylation Enhances the Immunogenic Potential of Tolinapant in T-cell Lymphoma
Source: Cancer Res Commun. 2024 Jun 6;4(6):1441–53. doi: 10.1158/2767-9764.CRC-23-0415 (PMC11155518; doi:10.1158/2767-9764.CRC-23-0415)
Supplement: Figure S6 — Additional Karpas-299 cytokine data. (Refers to Figure 4) [file crc-23-0415-s09.pptx]

## Slide 1
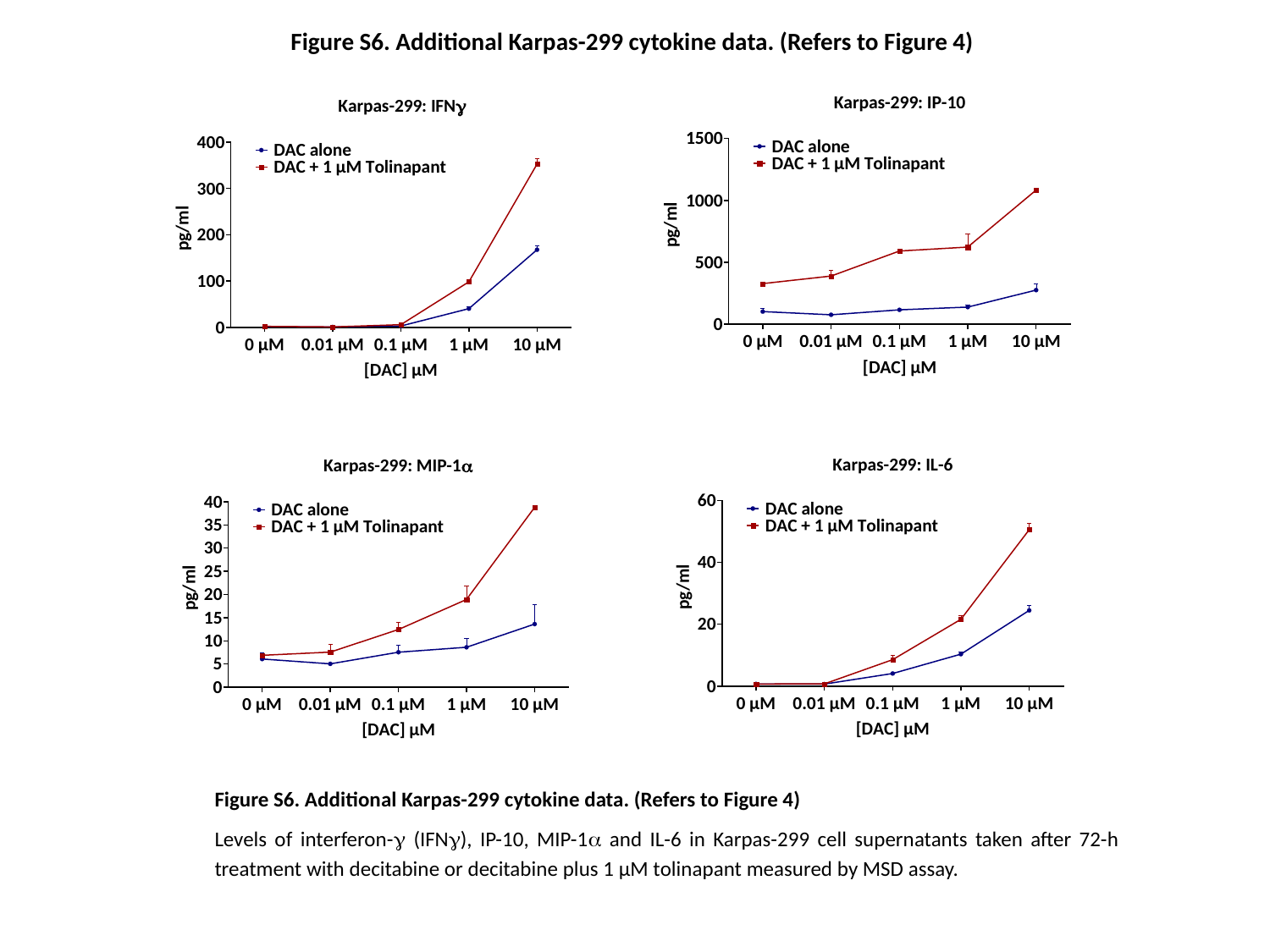

Figure S6. Additional Karpas-299 cytokine data. (Refers to Figure 4)
Figure S6. Additional Karpas-299 cytokine data. (Refers to Figure 4)
Levels of interferon-g (IFNg), IP-10, MIP-1a and IL-6 in Karpas-299 cell supernatants taken after 72-h treatment with decitabine or decitabine plus 1 µM tolinapant measured by MSD assay.
