# Supplementary material for: Epigenetic Priming by Hypomethylation Enhances the Immunogenic Potential of Tolinapant in T-cell Lymphoma
Source: Cancer Res Commun. 2024 Jun 6;4(6):1441–53. doi: 10.1158/2767-9764.CRC-23-0415 (PMC11155518; doi:10.1158/2767-9764.CRC-23-0415)
Supplement: Figure S7 — Additional in vivo PD data. (Refers to Figure 5) [file crc-23-0415-s10.pptx]

## Slide 1
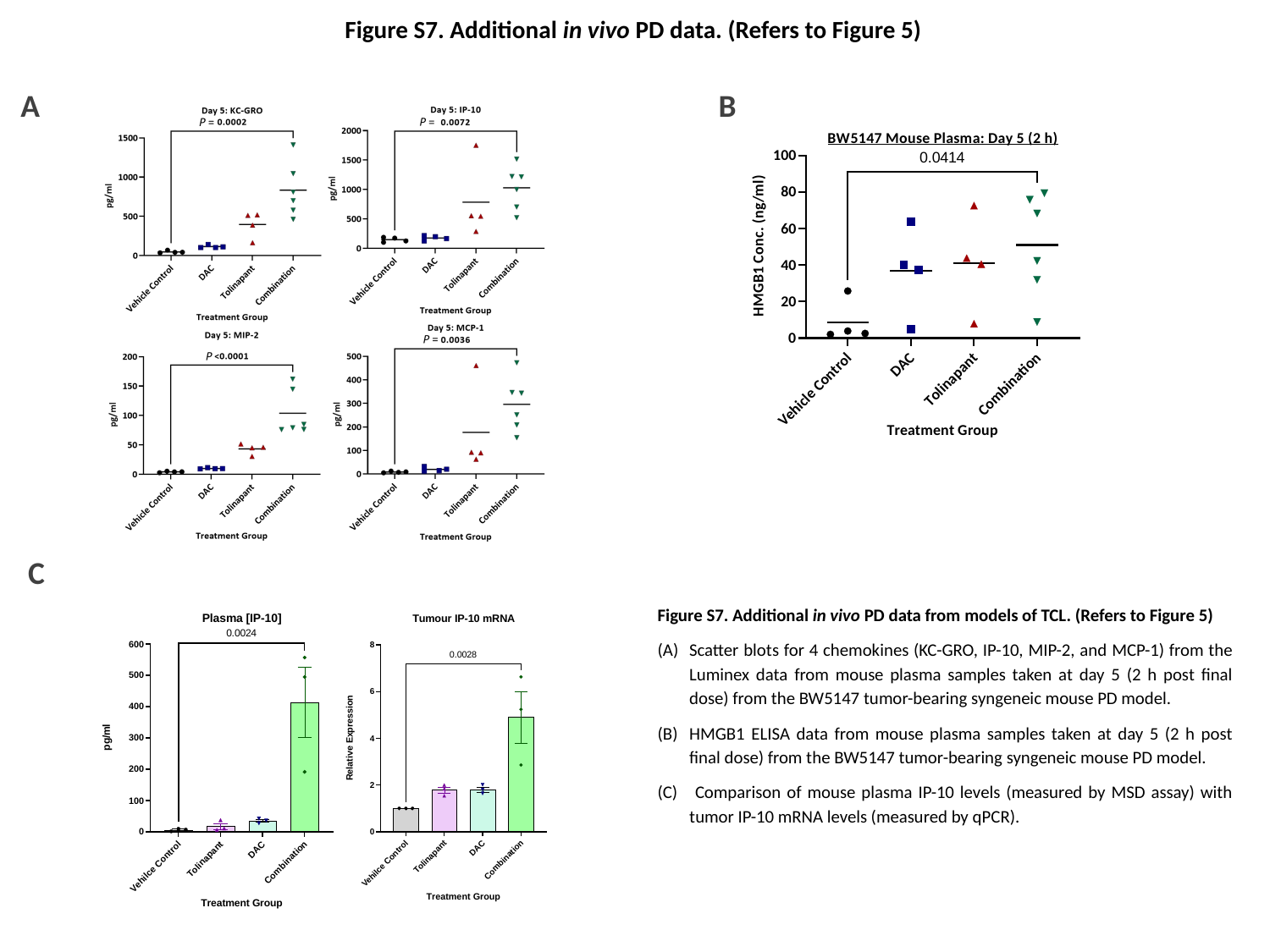

Figure S7. Additional in vivo PD data. (Refers to Figure 5)
A
B
C
Figure S7. Additional in vivo PD data from models of TCL. (Refers to Figure 5)
Scatter blots for 4 chemokines (KC-GRO, IP-10, MIP-2, and MCP-1) from the Luminex data from mouse plasma samples taken at day 5 (2 h post final dose) from the BW5147 tumor-bearing syngeneic mouse PD model.
HMGB1 ELISA data from mouse plasma samples taken at day 5 (2 h post final dose) from the BW5147 tumor-bearing syngeneic mouse PD model.
 Comparison of mouse plasma IP-10 levels (measured by MSD assay) with tumor IP-10 mRNA levels (measured by qPCR).
