# Supplementary material for: Epigenetic Priming by Hypomethylation Enhances the Immunogenic Potential of Tolinapant in T-cell Lymphoma
Source: Cancer Res Commun. 2024 Jun 6;4(6):1441–53. doi: 10.1158/2767-9764.CRC-23-0415 (PMC11155518; doi:10.1158/2767-9764.CRC-23-0415)
Supplement: Figure S8 — Additional EL4 & EL4-C8KO model in vivo efficacy data. (Refers to Figure 6) [file crc-23-0415-s11.pptx]

## Slide 1
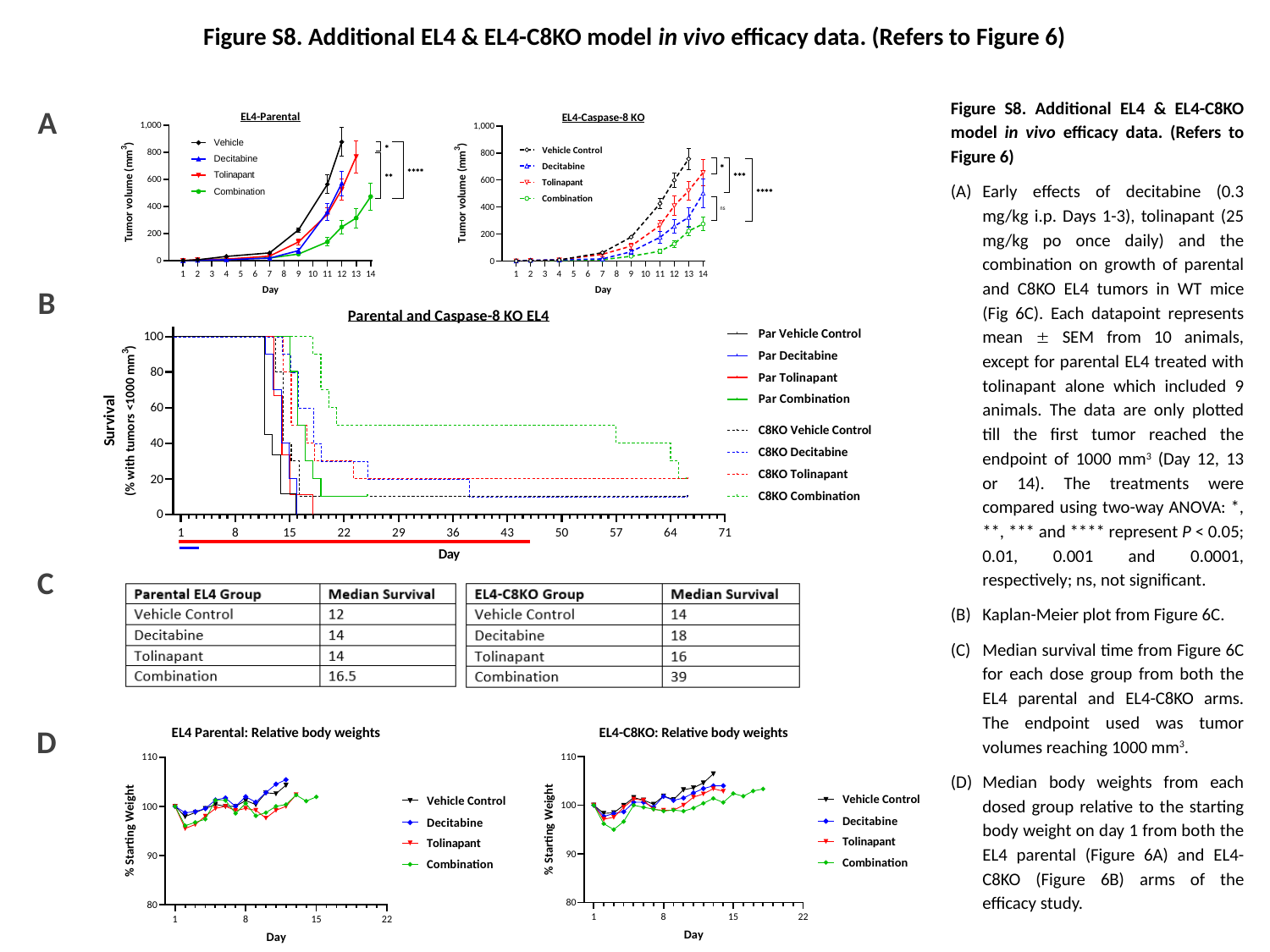

Figure S8. Additional EL4 & EL4-C8KO model in vivo efficacy data. (Refers to Figure 6)
# Figure S6:
Figure S8. Additional EL4 & EL4-C8KO model in vivo efficacy data. (Refers to Figure 6)
Early effects of decitabine (0.3 mg/kg i.p. Days 1-3), tolinapant (25 mg/kg po once daily) and the combination on growth of parental and C8KO EL4 tumors in WT mice (Fig 6C). Each datapoint represents mean ± SEM from 10 animals, except for parental EL4 treated with tolinapant alone which included 9 animals. The data are only plotted till the first tumor reached the endpoint of 1000 mm3 (Day 12, 13 or 14). The treatments were compared using two-way ANOVA: *, **, *** and **** represent P < 0.05; 0.01, 0.001 and 0.0001, respectively; ns, not significant.
Kaplan-Meier plot from Figure 6C.
Median survival time from Figure 6C for each dose group from both the EL4 parental and EL4-C8KO arms. The endpoint used was tumor volumes reaching 1000 mm3.
Median body weights from each dosed group relative to the starting body weight on day 1 from both the EL4 parental (Figure 6A) and EL4-C8KO (Figure 6B) arms of the efficacy study.
A
B
C
D
