# Supplementary material for: Epigenetic Priming by Hypomethylation Enhances the Immunogenic Potential of Tolinapant in T-cell Lymphoma
Source: Cancer Res Commun. 2024 Jun 6;4(6):1441–53. doi: 10.1158/2767-9764.CRC-23-0415 (PMC11155518; doi:10.1158/2767-9764.CRC-23-0415)
Supplement: Figure S9 — Analysis of tumor and plasma samples from EL4-Parental and EL4-C8KO syngeneic model PD studies (Refers to Figure 6) [file crc-23-0415-s12.pptx]

## Slide 1
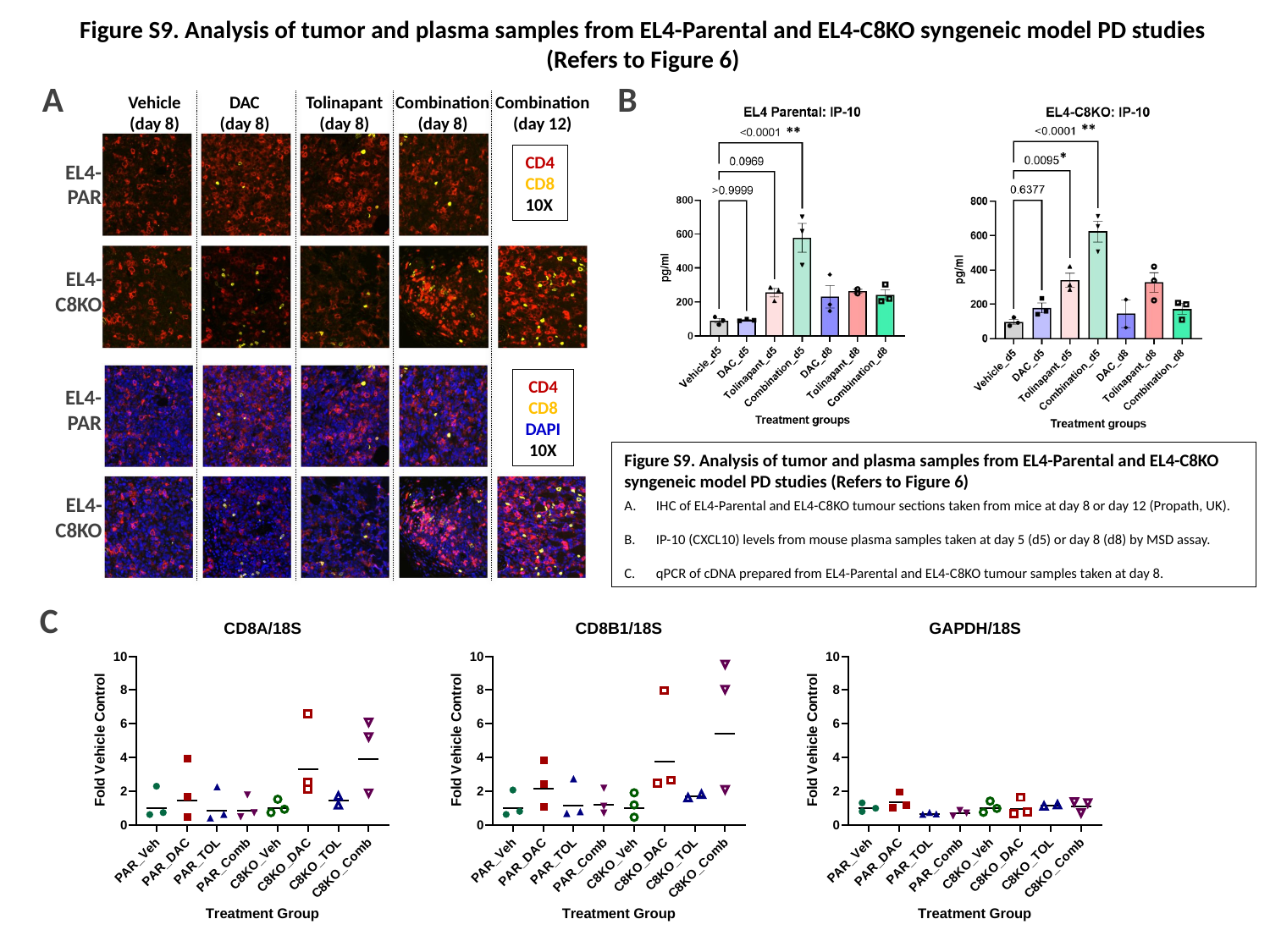

Figure S9. Analysis of tumor and plasma samples from EL4-Parental and EL4-C8KO syngeneic model PD studies
(Refers to Figure 6)
A
B
Vehicle
(day 8)
DAC
(day 8)
Tolinapant
(day 8)
Combination
(day 8)
Combination
(day 12)
CD4
CD8
10X
EL4-
PAR
EL4-
C8KO
CD4
CD8
DAPI
10X
EL4-
PAR
Figure S9. Analysis of tumor and plasma samples from EL4-Parental and EL4-C8KO syngeneic model PD studies (Refers to Figure 6)
IHC of EL4-Parental and EL4-C8KO tumour sections taken from mice at day 8 or day 12 (Propath, UK).
IP-10 (CXCL10) levels from mouse plasma samples taken at day 5 (d5) or day 8 (d8) by MSD assay.
qPCR of cDNA prepared from EL4-Parental and EL4-C8KO tumour samples taken at day 8.
EL4-
C8KO
C
