# Supplementary material for: Epigenetic Priming by Hypomethylation Enhances the Immunogenic Potential of Tolinapant in T-cell Lymphoma
Source: Cancer Res Commun. 2024 Jun 6;4(6):1441–53. doi: 10.1158/2767-9764.CRC-23-0415 (PMC11155518; doi:10.1158/2767-9764.CRC-23-0415)
Supplement: Figure S10 — Impact of tolinapant and decitabine on induction of immunogenic cell death in TCL. (Refers to Discussion) [file crc-23-0415-s13.pptx]

## Slide 1
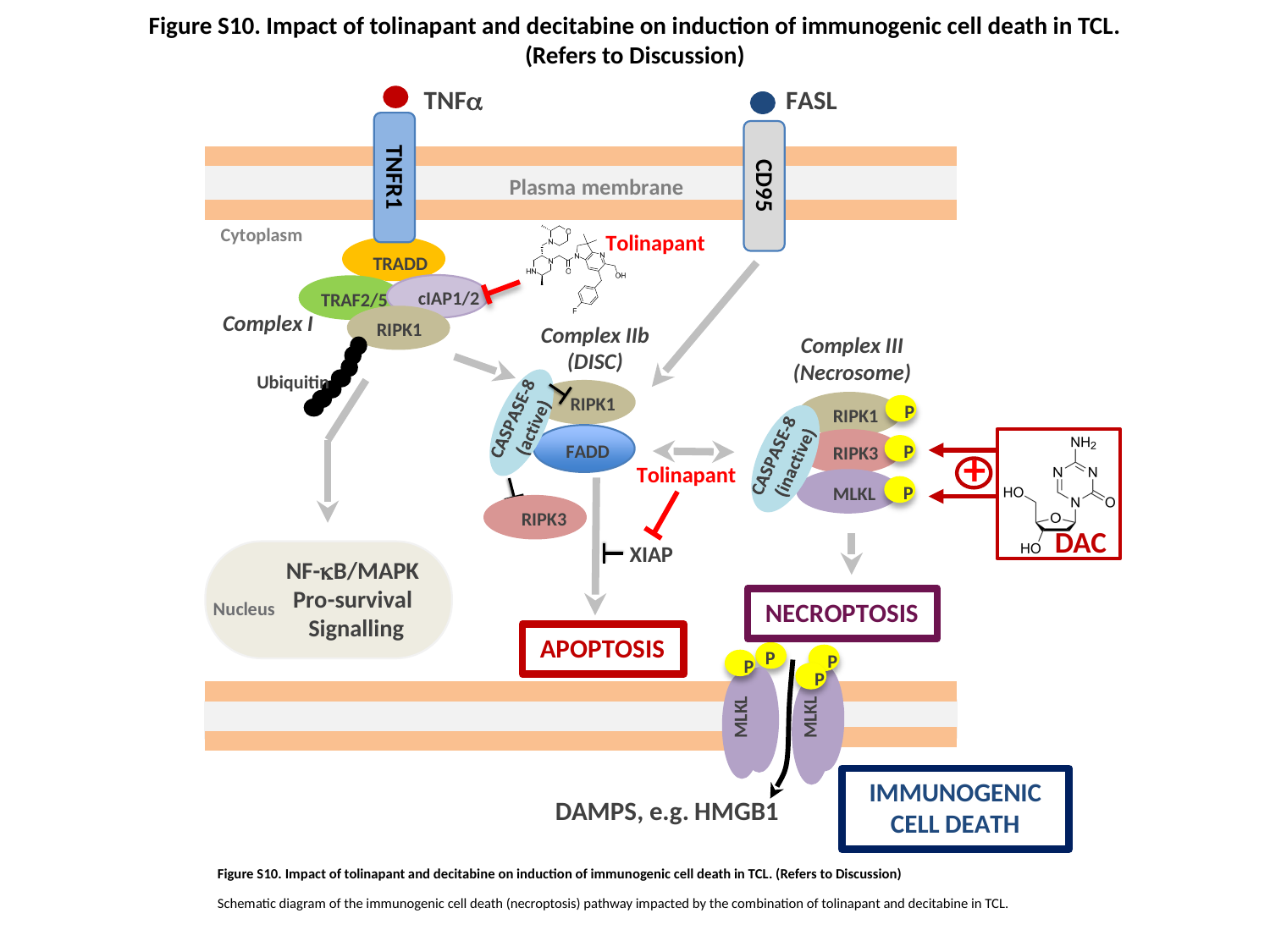

Figure S10. Impact of tolinapant and decitabine on induction of immunogenic cell death in TCL.
(Refers to Discussion)
Figure S10. Impact of tolinapant and decitabine on induction of immunogenic cell death in TCL. (Refers to Discussion)
Schematic diagram of the immunogenic cell death (necroptosis) pathway impacted by the combination of tolinapant and decitabine in TCL.
