# Supplementary material for: Epigenetic Priming by Hypomethylation Enhances the Immunogenic Potential of Tolinapant in T-cell Lymphoma
Source: Cancer Res Commun. 2024 Jun 6;4(6):1441–53. doi: 10.1158/2767-9764.CRC-23-0415 (PMC11155518; doi:10.1158/2767-9764.CRC-23-0415)
Supplement: Figure S11 — Uncropped Western blots from Figure 2A [file crc-23-0415-s14.pptx]

## Slide 1
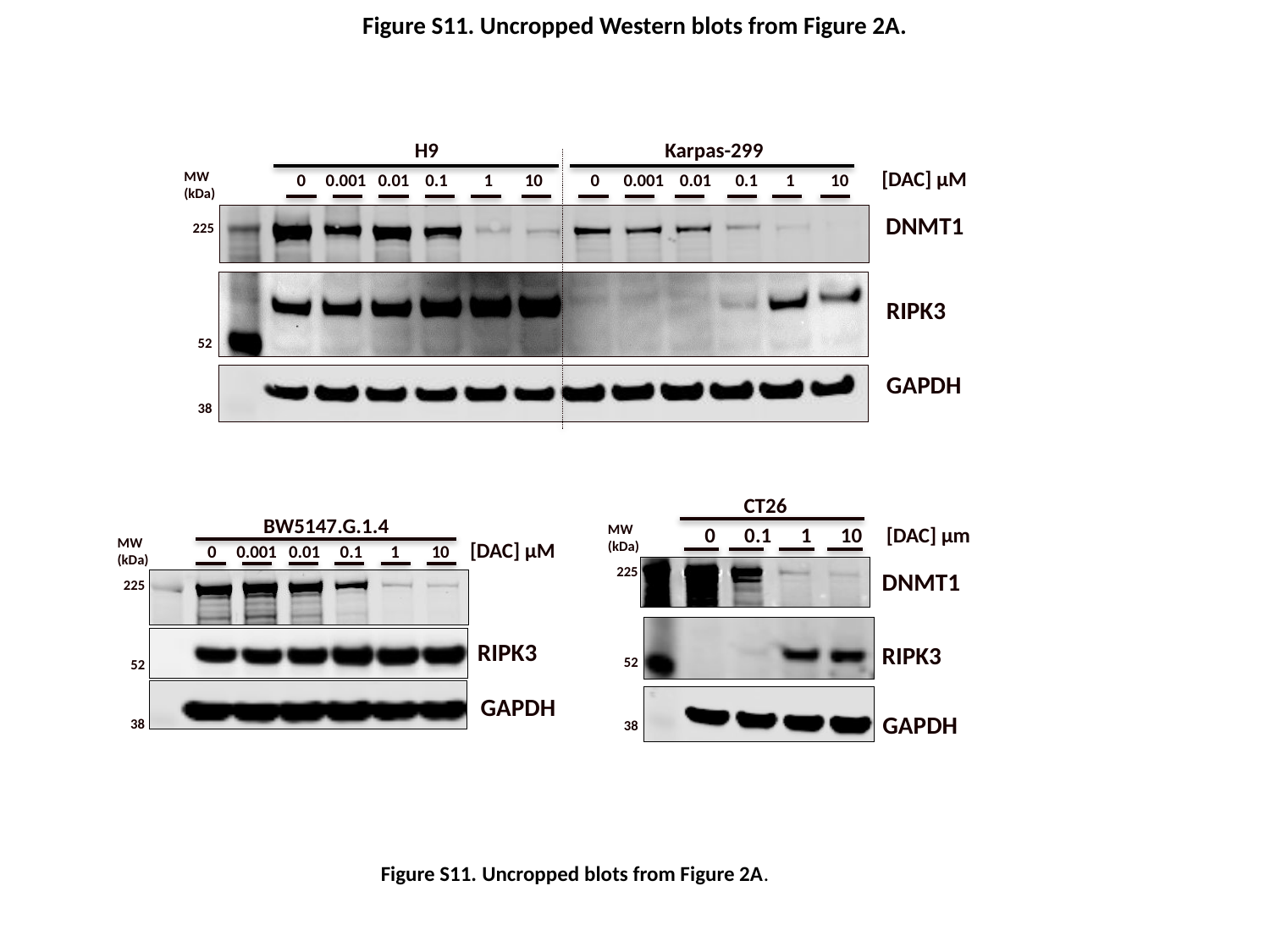

Figure S11. Uncropped Western blots from Figure 2A.
H9
Karpas-299
0 0.001 0.01 0.1 1 10 0 0.001 0.01 0.1 1 10 [DAC] µM
MW
(kDa)
DNMT1
225
RIPK3
52
GAPDH
38
CT26
BW5147.G.1.4
MW
(kDa)
 0 0.1 1 10 [DAC] µm
MW
(kDa)
0 0.001 0.01 0.1 1 10 [DAC] µM
225
DNMT1
225
RIPK3
RIPK3
52
52
GAPDH
GAPDH
38
38
Figure S11. Uncropped blots from Figure 2A.
